# Using voice and speech data in healthcare: a scoping review of the ethical, legal and social implications

**DOI:** 10.3389/fdgth.2026.1750111

**Published:** 2026-02-10

**Authors:** Marie-Françoise Malo, Sarah Bouhouita-Guermech, Hortense Gallois, Vardit Ravitsky, Bridge2AI Voice Consortium, Jean-Christophe Bélisle-Pipon

**Affiliations:** 1 Faculty of Health Sciences, Simon Fraser University, Burnaby, BC, Canada; 2 École de Santé Publique, Université de Montréal, Montréal, QC, Canada; 3 Centre of Genomics and Policy (CGP), McGill University, Montréal, QC, Canada; 4 The Hastings Center for Bioethics, Garrison, NY, United States

**Keywords:** artificial intelligence, biomarkers, ELSI, ethics, speech, voice biomarkers, voice

## Abstract

Human voice and speech, integral to personal identity and social communication, are increasingly used as biometric and digital biomarkers in healthcare. Their collection and analysis, enabled by artificial intelligence, machine learning, and natural language processing, offer promising applications in disease detection and health monitoring. This scoping review examines the ethical, legal, and social implications (ELSIs) associated with using voice and speech data in healthcare. Following a structured search of four databases and a snowball method, 65 articles published between 2009 and 2024 were analyzed. The findings are organized into three main ELSI categories: ethical concerns include privacy breaches, challenges of informed consent, and the need for data validation and respect for vulnerable populations; social issues highlight biases, representational disparities, and risks of discrimination and data misuse; legal issues include unclear regulatory frameworks, conflicting jurisdictional mandates, and challenges in defining data ownership. The review reveals that while many ELSIs mirror those of other biomarker data, the unique properties of voice and speech require adapted frameworks for consent, data governance, and privacy protection. Technological limitations, dataset scarcity, and industry-academic divides exacerbate risks and hinder equitable development. Few studies deeply explore ELSIs in underrepresented populations, and there is a lack of robust empirical research. The review argues for a contextualist, not exceptionalist, approach to voice biomarkers, acknowledging both overlapping and unique challenges. It concludes by stressing the need for harmonized regulations, inclusive datasets, and interdisciplinary collaboration to ensure responsible, equitable integration of voice and speech technologies in healthcare.

## 1 Background

The production of human voice and speech is an intricate process that involves the coordination of respiration, phonation, articulation, resonation and prosody (1, 2) and depends on the collaboration of several motor and cognitive processes (2, 3) and more than 100 muscles (4).^1^ These processes are directly impacted by human physiological features. For example, the length and shape or human vocal tract and the dynamic configuration of articulatory organs can directly affect sound and speech production (7). Voice and speech enable humans to convey their moods and assert their personality (8), communicate with their environment, express emotions, and detail thoughts (9–11). Speech and voice, through both their physiological basis and social use, shape auditory identity, serving as an “invisible business card” presented to the world (12).

In recent years, these characteristics have led to the popularization of using vocal biometrics for tasks like voice authentication, speaker detection, and forensic speaker recognition (13). The uniqueness of voice and speech has even been compared to that of fingerprints (14), although some authors have contested this comparison (15). Recording voice and speech is quick, inexpensive, non-invasive, and can be done remotely (14–17). Furthermore, recognizing people using their voice and being recorded are familiar processes, especially as microphones have become more ubiquitous in modern day life (18).

Aided by the developments in the field of voice biometrics and the successes of data-driven approaches for consumer speech applications, there has been a recent surge in interest in leveraging human voice and speech as sources of data for healthcare. Voice and speech biomarkers, derived from the nuances in voice and speech, represent a powerful yet underused resource in healthcare (19). Traditionally, biomarkers have been described as “a defined characteristic that is measured as an indicator of normal biological processes, pathogenic processes, or biological responses to an exposure or intervention, including therapeutic interventions” (20). Biomarkers can have different putative applications, ranging from prediction, diagnostic, or monitoring of a condition or a treatment, to the study of pharmacodynamic responses to medications. Voice biomarkers are a part of the new frontier known as digital biomarkers (21), where behavioural and physiological data is collected using digital devices to aid in diagnosis, monitoring, and treatment of various health conditions (22, 23). Although the term digital biomarker has recently been under some scrutiny for being misleading, unclear, and ill-defined (24–26), no other common term has emerged or gained consensus among scholars. Voice biomarkers are defined as distinct vocal attributes, alone or in combination, that have been scientifically validated as indicators of clinical outcomes (27, 28). They can be used for symptom detection and monitoring, diagnosis of conditions, screening potential health issues, perceived health status monitoring, and to monitor disease progression (29). Similar to its use as biometric information, voice biomarkers have the advantage of being easy and relatively inexpensive to collect and preserve in databases (30, 31).

Even though the idea of using voice as a biomarker of health has been explored for decades (32), recent developments in artificial intelligence (AI), machine learning (ML), and natural language processing (NLP) have enabled fast, precise analysis of voice data, making it possible to envision the scalable development of voice biomarkers analysis (33–35). Voice biomarkers have already demonstrated their potential in the diagnosis of diseases and conditions such as Alzheimer's disease (36), Parkinson's disease (37), diabetes, developmental problems such as autism (38), and various mental health problems such as depression, stress, and suicidal ideation (39, 40). However, there are a limited number of voice datasets meant for clinical use (41). These datasets are often incomplete, and due to this fact researchers often lack the capacity to consider implications of diversity and privacy control (42) or properly train algorithms with them (27). This has led to calls to curate large, scalable, standardized databases to use for research on voice and speech biomarkers (34, 43, 44).

While work has been done on the ethical, legal, and social implications (ELSI) of using voice biometrics (45–48), the collection of voice and speech for clinical use requires a deeper understanding of the ELSI specific to this nascent use (44, 49). This scoping review aims to present the state of the literature on the ELSI that arise from the collection and use of voice and speech data in healthcare, while also adding to the growing literature on the ELSI of digital biomarkers (50–53). Moreover, it aims to identify how the collection of voice and speech data for medical and clinical use can redefine some of the ELSI related to digital biomarkers, how it fits with current understanding of the ELSI related to biomedical and clinical data, and to address the gaps in the existing literature and potential areas of concerns. The aim of this analysis is to contribute to the responsible development and use of voice and speech biomarkers.

## 2 Method

A scoping review was realized to appraise and determine the volume of literature and studies available on the ELSI of the use of voice as a biomarker of health (54, 55). A scoping review approach was selected because it allows for a comprehensive mapping of the diverse studies and disciplines addressing the evolving challenges posed by voice data in healthcare. Rigor was ensured by engaging in a predefined and comprehensive search strategy (56, 57). This scoping review was conducted in 3 phases. A first phase (Phase 1) consisted in finding articles through a search on databases, a second phase (Phase 2) was added where articles were found through snowball research from Phase 1, and a third phase (Phase 3) was conducted to review new literature. Each phase, and its associated steps, are described below. The charting of the data and the collating, summarizing and reporting of results are presented in the Results section.

### 2.1 Phase 1: initial database search

References from this review of the literature were identified through Ovid MEDLINE, Web of Science, Ovid EMBASE and IEEE. Ovid Medline and Ovid Embase were selected to ensure comprehensive coverage of healthcare-related literature, while IEEE Xplore and Web of Science were included to capture engineering perspectives and broader multidisciplinary research. Search terms were categorized into three main themes: *Ethical, legal* or *social implications*, *voice* or *voice as a biomarker,* and *medical technologies.* Established themes were populated using MeSH terms, Emtree terms, Boolean operators, and informed by key search literature. Discussions with experts within the Bridge2AI-Voice Consortium informed the selection of relevant terms relevant to voice biomarkers and voice health, and a university librarian specialized in scoping reviews helped adapt the search strategy to the scope of the database. Table 1 presents the terms that were used in the different databases.

**Table 1:** Search strategy.

Concepts	Terms
ELSI	**MD** **=** ((Ethic* or Bioethic* or Moral* or ELSI or ELSA or ((social or bioethical or ethical) adj (issue* or aspect* or impact* or consequence* or implication* or “effect” or “effects” or consideration* or challenge*))).mp. or informed consent/ or Bioethical Issues/ or Ethics/) not “ethics committee”.mp. not “ethical approval”.mp. not “ethics and dissemination”.mp. **EMB =** ((Ethic* or Bioethic* or Moral* or ELSI or ELSA or ((social or bioethical or ethical) adj (issue* or aspect* or impact* or consequence* or implication* or “effect” or “effects” or consideration* or challenge*))).mp. or informed consent/ or Bioethical Issues/ or Ethics/) not “ethics committee”.mp. not “ethical approval”.mp. not “ethics and dissemination”.mp. **WoS =** Ethic* OR Bioethic* OR Moral* OR “ELSI” OR “Public NEAR/0 Policy” OR “informed NEAR/0 consent” OR “social NEAR/0 responsibilities” OR “Data NEAR/2 anonym*” OR “medical NEAR/0 ethics” OR ((social or bioethical or ethical) NEAR/3 (issue* OR aspect* OR impact* or consequence* OR implication* OR effect* OR consideration* OR challenge* OR policy OR policies)) (Topic) **IEEE =** Ethic* OR Bioethic* OR Moral* OR ELSI OR ELSA OR “social issue” OR “social aspect” OR “social impact"
Voice	**MD** **=** (Voice or Vocal or Acoustic or Speech or Bioacoustics or Respirat* or Phonation or Resonation or Articulat*).mp. **EMB =** (Voice or Vocal or Acoustic or Speech or Bioacoustics or Respirat* or Phonation or Resonation or Articulat*).mp. **WoS =** Voice OR Vocal OR Speech OR “conversation* NEAR/3 agent*” OR Acoustic OR Bioacoustics OR Respirat* OR Phonation OR Resonation OR Articulat* IEEE = Voice OR Vocal OR Speech OR Acoustic
Health Technology	**MD** **=** [Technolog* or Informatic* or Biomarker* or (“health care” adj1 technology)].mp. **EMB =** [Technolog* or Informatic* or Biomarker* or (“health care” adj1 technology)].mp. **WoS =** Technolog* OR Informatic* OR Biomarker* OR Diagnostic* (Topic) **IEEE =** Technology OR Technologies OR Informatic OR “machine learning” OR “algorithm” OR “natural language processing” OR “artificial intelligence” OR biobank OR dataset
Legend	MD = MedLine; EMB = Embase; WoS = Web of Science; IEEE = Institute of Electrical and Electronics Engineers

The initial database search was carried out on December 14, 2022, for all four databases, and 2,393 articles extracted were uploaded into Covidence (58). Three hundred eighty-four (384) duplicates were removed. For the first screening, the team outlined a list of inclusion criteria: articles were included if the appraisal of their title and abstract met the following criteria: (1) written in English or French; (2) defined as a peer-reviewed article, commentary, editorial, review, or discussion paper; and (3) included topics that intersected the three main themes from the literature review. No restrictions were placed on the date of publication, design, or date of publication. Three screeners persons (MFM, SBG, QH) independently applied the inclusion and exclusion criteria on the 2,009 remaining papers (59). In instances of disagreement, consensus was achieved through discussion between the three reviewers, with advice from someone with experience in literature reviews (JCBP).

The title and abstract screening process removed 1,899 papers from the scoping review at hand. Full-text review was then done to assess the eligibility of the 110 remaining papers. Following exclusion criteria were used to sort and evaluate studies: (1) were not English or French; (2) did not address questions ELSIs; (3) did not relate to voice or speech; (4) did not relate to voice data collection or voice data usage in technology; (5) were not healthcare related; (6) book sections or chapters; (7) had no full text available. The same screeners (MFM, SBG, QH) independently applied the exclusion criteria. Again, disagreements were discussed between reviewers until consensus was achieved. In the end, 24 studies were selected, with advice from the principal investigator (JCBP).

### 2.2 Phase 2: snowball search

Considering the small number of articles included in the scoping review and the key aim of a scoping review to assess the extent of discussion around a topic, the research team (MFM, SBG, JCBP), following the recommendations of a university librarian specialized in scoping reviews, carried out a second round of research snowballing from the 24 included papers. Two members of the team (MFM, SBG) retrieved all the papers cited in the 24 papers, as well as all the articles citing these included papers. This work added 1,650 articles to the original search. A second round of title, abstract, and full-text screening was carried out on these articles, following the same criteria identified above. At the end of this process, 24 additional papers were included increasing the total number of studies in the scoping review to 48.

### 2.3 Coding of the results from phase 1 and phase 2

Two articles were coded using NVivo 14 (60) by members of the team (MFM, SBG) to create the initial coding frame (61). The coding frame was developed around 5 overarching themes: (1) voice as a biomarker; (2) ethical implications; (3) social implications; (4) legal implications; (5) mitigating solutions. The coding frame was reviewed by an experienced coder (JCBP), who provided comments and guidance. During the coding process, members of the team (MFM, SBG) reviewed two papers with the initial coding frame to assess the degree of intra-coder agreement. Two coders (MFM, SBG) reviewed the remaining data individually. New codes were inductively added to the initial coding frame based on the content of the articles. Weekly meetings between the two coders were organized to ensure agreement between coders. After coding, the two coding files were merged to create a final coding grid. Each article was reviewed with the final coding grid to ensure a consistent coding.

### 2.4 Phase 3: subsequent database search and analysis of results

**Figure 1:**
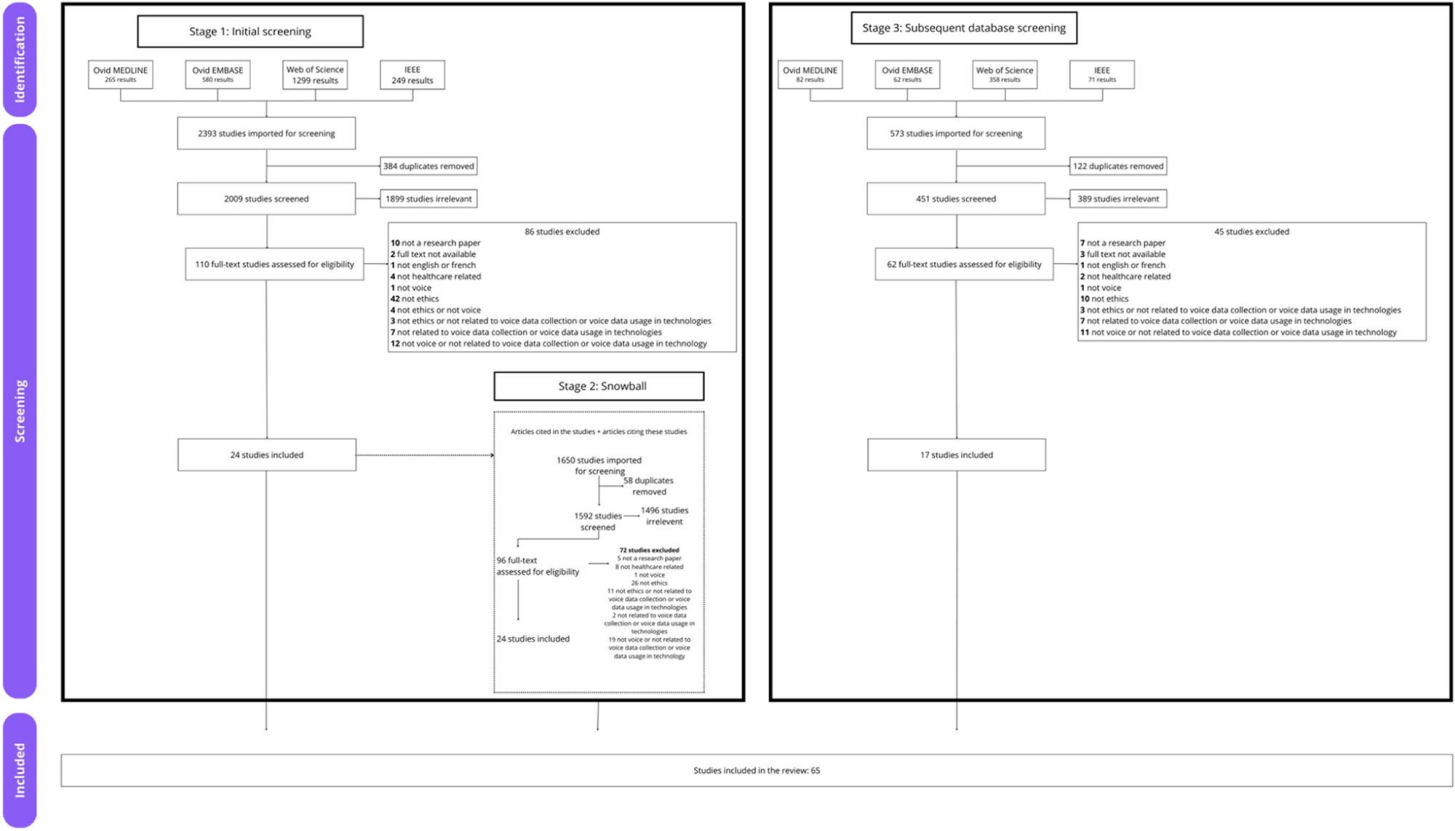
Prisma flowchart of search outcomes.

A second search of the previously identified databases with the same terms was carried out in April 2024 to review articles published since the initial search in December 2022. Following this, the articles found were uploaded to Covidence and two team members (MFM, AG) used the same inclusion criteria to sort through the title and abstract and through the full text. In total, 413 articles were imported for title and abstract screening, 43 full-text articles were reviewed, and 17 articles were included, bringing the total number of articles in the review to 65. Figure 1 presents our review flowchart following PRISMA's guidelines (62) for all three stages. Papers were thematically analyzed using NVivo 14 (60). The initial coding grid used was the final coding grid from stage 2.3. During the coding process, members of the team (MFM, AG) reviewed two papers with the coding grid to assess the degree of intra-coder agreement. Research team members (MFM, AG) reviewed the remaining data individually. New codes were inductively added by MFM and AG to the coding grid based on the content of the articles. Weekly meetings between the two coding team members were organized to ensure agreement between coders. After coding, the two coding files were merged to create a final coding grid. Each article (from the first and subsequent database search) was reviewed with the final coding grid to ensure a consistent coding.

## 3 Results

Results are presented following the different themes of the coding. Each of the three types of implications (ethical, social, and legal) are examined in two ways. The first category, “conventional implications”, covers issues arising from the use of voice and speech data in healthcare that parallel those linked to other biometric data, such as gait or ocular movement analysis (63, 64), and defines where voice and speech are treated as continuous with existing health data ethics discussions. The second category, “modality-specific implications”, addresses ELSI that require refinement or revision due to the unique characteristics of voice and speech data, and defines where voice and speech data are challenging conversional frames.

As noted, not all of the included articles were specifically related to voice biomarker research or the use of voice biomarkers in healthcare. Although they addressed voice biomarker research and use, some articles dealt with digital biomarker research more generally, the use of smartphones or sensors in healthcare, or new trends in the treatment of a specific disease. Contrasting these implications with those brought on specifically by voice and speech data makes it possible to recognize the distinct risks and responsibilities associated with voice, while maintaining coherence across wider conversations on health data governance. In each of those categories, the implications are presented in order of how frequently they were mentioned by authors.

### 3.1 Overview of results

**Figure 2:**
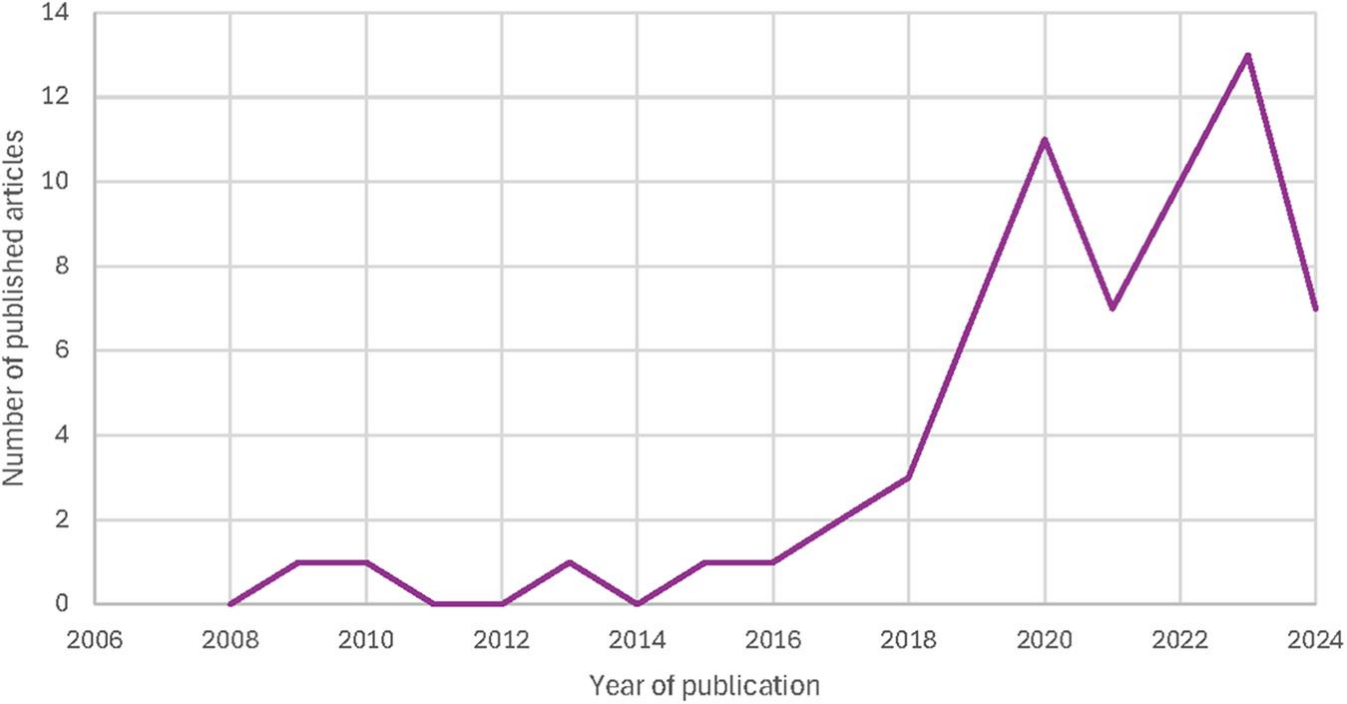
Number of included articles published per year (2008-2024).

**Figure 3:**
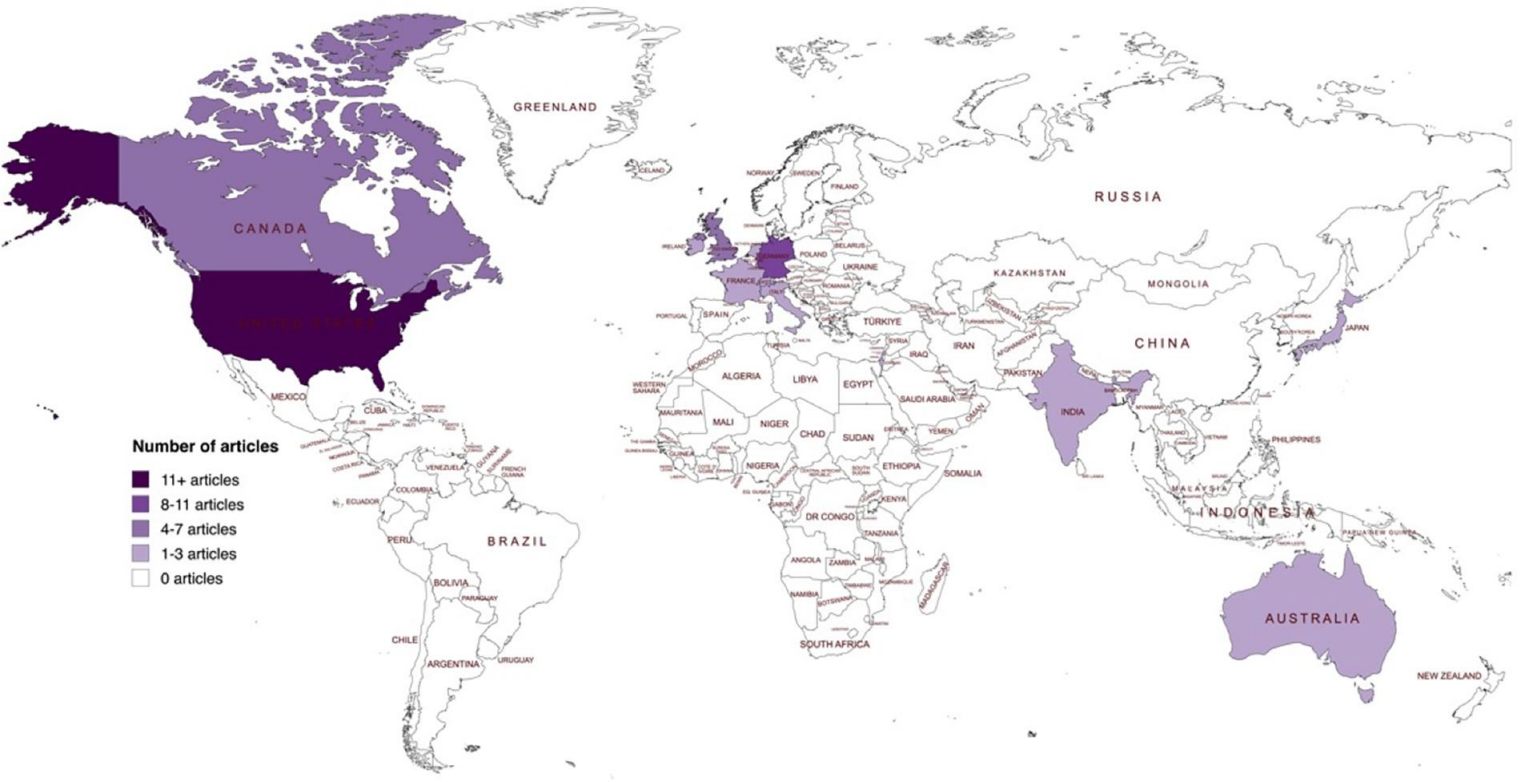
Geographic distribution of included articles based on the country of the first author or research team. This map illustrates the global distribution of the 67 articles included in the review. The majority originated from the United States of America (*n* = 27), followed by Germany (*n* = 8), Canada and the United Kingdom (*n* = 5 each), Switzerland and Australia (*n* = 3 each), and several other countries contributing one or two publications each (Italy = 2, Greece = 1, India = 2, Taiwan = 1, Colombia = 1, Netherlands = 2, Ireland = 2, France = 2, Japan = 1, Israel = 1, Luxembourg = 1). Countries are shaded according to the number of publications, with darker tones indicating a higher number of articles. World map created using MapChart (https://www.mapchart.net/), licensed under CC BY-SA 4.0.

**Figure 4:**
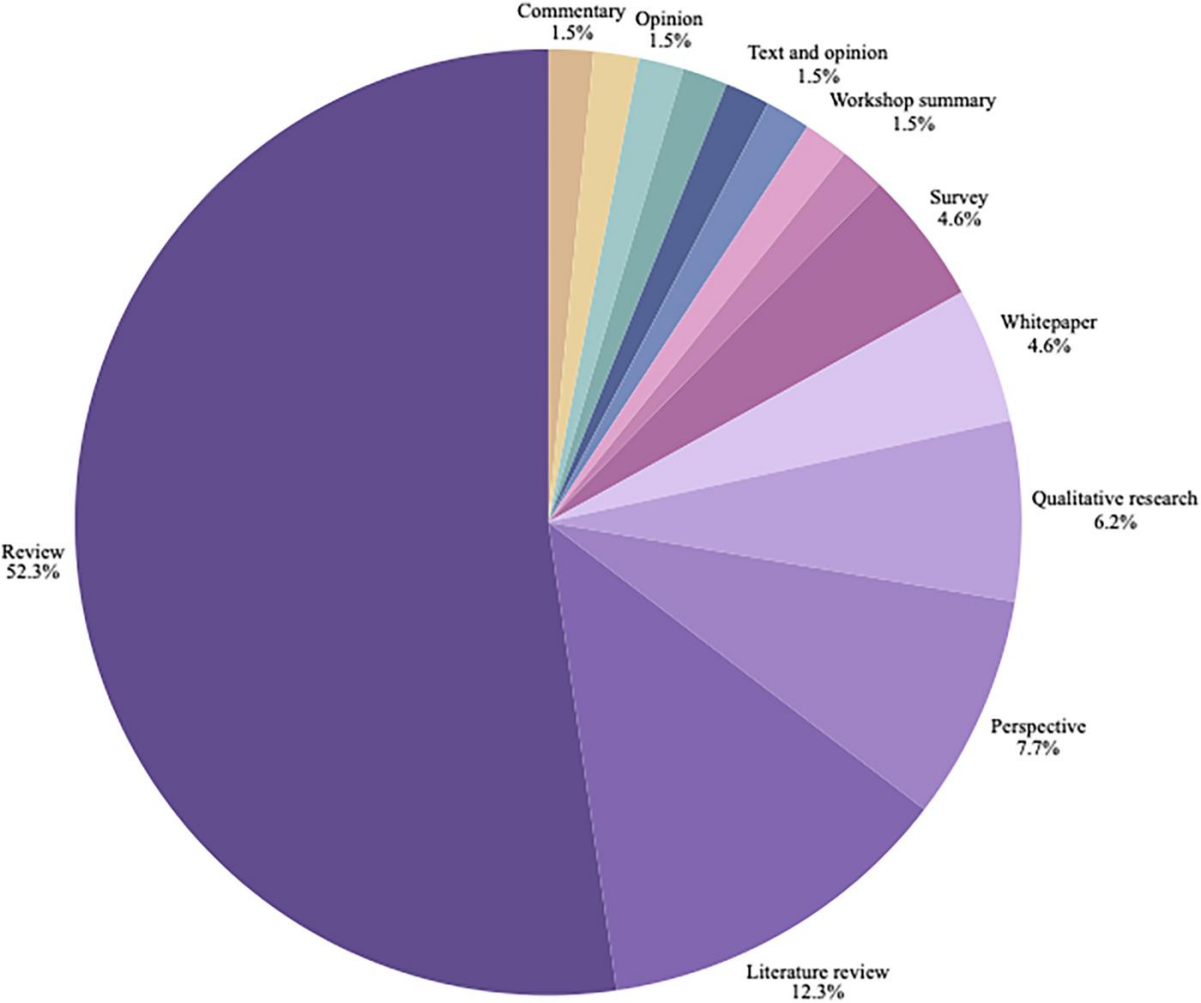
Publication types of included articles. The figure displays the distribution of article types, ordered by frequency: reviews (*n* = 34), literature reviews (*n* = 8), perspectives (*n* = 5), qualitative research articles (*n* = 4), surveys (*n* = 3), whitepapers (*n* = 3), and single-publication categories including commentary, longitudinal waitlist-control field study, opinion, position paper, quantitative study, text and opinion, viewpoint, and workshop summary (*n* = 1).

**Figure 5:**
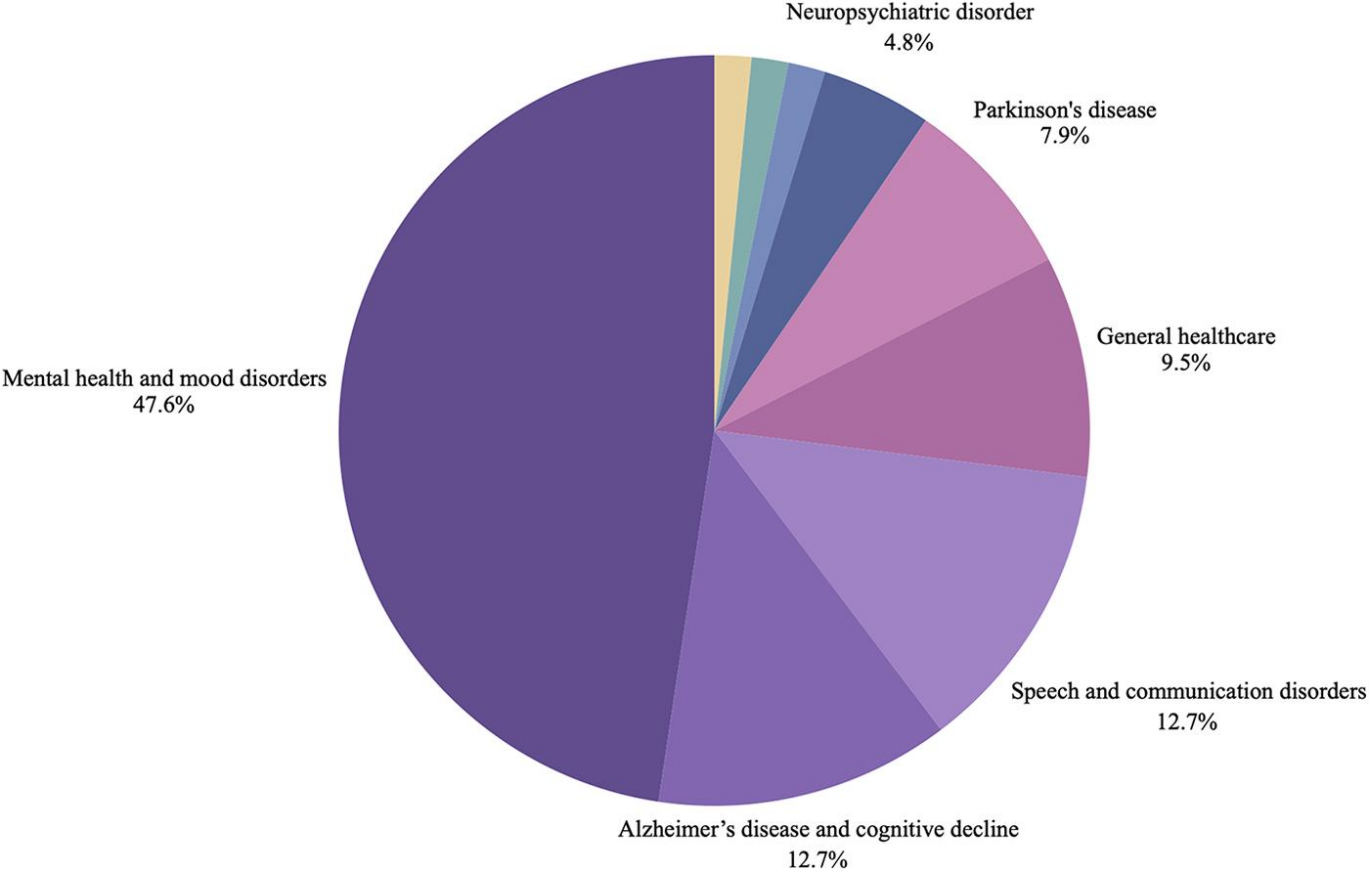
Field of health research of included studies. The figure presents the distribution of health conditions examined across the included papers, ordered by frequency: mental health and mood disorders (*n* = 30), speech and communication disorders (*n* = 8), Alzheimer's disease and cognitive decline (*n* = 8), general healthcare applications (*n* = 6), Parkinson's disease (*n* = 5), neuropsychiatric disorders (*n* = 3), and single-paper categories including COVID-19, heart failure, and Huntington's disease (*n* = 1).

The 65 included articles were published between 2009 and 2024, with most of the articles published between 2019 and 2024 from teams in different parts of the world. Figure 2 presents a timeline of the number of included publications per year. Figure 3 presents a world map of the different countries of either the first author or the research teams. About half of the articles (*n* = 34) were reviews (e.g., scoping review, systematic review, contemporary review). Figure 4 presents a pie chart of the different types of articles included in this review. They touched on a variety of diseases, ranging from mental health conditions and behavioral health to Alzheimer's disease, Parkinson disease, heart failure, Covid-19, and speech disorders. Figure 5 presents the field area of included articles. All selected publications included elements related to the use or interpretation of voice and speech data in healthcare or research settings. As mentioned previously, to be included, publications were required to meaningfully engage with the topics of voice and speech biomarkers, whether in a dedicated section or as part of a wider discussion. In practice, this engagement varied across the literature: in some publications, voice biomarkers constituted the main focus of the research, with detailed conceptual, technical, or clinical discussions; in others, they appeared more tangentially, often mentioned as emerging tools within larger explorations of artificial intelligence, digital phenotyping, or data-driven health approaches.

### 3.2 Ethical implications

#### 3.2.1 Conventional implications

##### 3.2.1.1 Privacy and security

A number of publications report privacy issues arising from continuous, passive collection of voice and speech data (65–68). Privacy is a major concern for any remote monitoring approach (69), as the guarantees on privacy protections that can be provided are limited (70) and because privacy has no formal legal definitions (71). However, privacy regulations have been described as stifling to technological innovations (65, 72).

Articles highlight two main types of considerations. First, continuous data sharing is required to capture the amount of data accumulated through continuous and passive data-collection methods, which increases the risk of privacy breaches during this process (67, 73–75). Storing large amounts of data can also increase the risk of a privacy breach or data leakage, and also the likelihood of data being sold in the secondary data market (69, 70, 72, 74, 76–79). Second, continuous recording can also quickly become perceived as surveillance (80). One participant in a study from Hurley & al (76). mentioned that constant data collection on any type of data is “overkill”. In the same study, another participant described it as a form of “personal intrusiveness”. This can apply to the patients or the subject being recorded, but also to their caregivers as well who may also have their own concerns over monitoring (81). To mitigate this concern, Popp & al (82)., in a review on the shift from active to passive monitoring of Alzheimer's disease, mention that mandating that a person interact with a device can also represent a conscious effort on people's part to remind them that they are being recorded and lessen the impression of passive surveillance and loss of control, especially for people with chronic or degenerative condition that affect their own sense of control (72). Similarly, Bailey, Patel & Guari (83), while focusing on data collection involving minors, raise a broader concern: the trade-off between gathering more data to train models effectively and maintaining privacy protections. This tension is relevant across all populations, particularly when sensitive data like voice is involved.

Voice and speech data is also potentially identifiable and can be used for multiple nefarious undertakings. For example, this data can be used to surveil individuals and intrude on their private lives (73, 76, 80), especially if voice data provides a window in an individual's thoughts (76). That private information could then be used by advertising companies to target individuals in a compromised emotional state (68, 72, 79, 84). One article also mention the potential for mass surveillance of whole sub-groups of the population (85).

Research on voice and speech data also sometimes requires and benefits from the sharing of data in publicly available repositories (70, 86) or with other research groups (87). Ensuring the security of the data when sharing with other research groups is a specific concern raised by one article (81). With respect to privacy, participants have different expectations regarding data access and data sharing (81, 88, 89), and even if authors mention that there should be a clear understanding in consent forms regarding the types of data that will be shared, to whom, how, and why (70, 90), there seems to be a lack of understanding of what people want regarding their privacy in voice processing (91). These concerns also apply to data collection done for research, as well as data collected for clinical use. However, privacy protection can be harder to sustain in smaller research projects, because the identity of the subjects is often known (92). Sharing voice data to a new group of researchers can also lead to re-identification of subjects because data subjects via other means, for example with cross-identification with other datasets (75).

##### 3.2.1.2 Informed consent

A study by Hurley et al. on multimodal computer perception and neurotechnology mentions that, for data collections on new types of data like voice, it can be hard, at the time of consent, to explain to people and patients how they can be harmed if their data is leaked to unwanted third parties and how that can lead to long-term risks that are difficult to anticipate (76). This can affect the transparency towards participants, who must understand what data is collected, when and where (70, 75). Additionally, participants' level of literacy (general and technical) can affect how well they grasp the content of consent forms (66, 90). Information on data collection, storage, and usage should thus be written in language that is appropriate and adapted to the group of participants (70, 75, 90).

##### 3.2.1.3 Accessibility of data

Some articles mention that voice data sharing between research groups can be difficult on a practical level. For example, voice data (or their derivatives) can be stored in a format that is not convenient or transmittable, or else different research projects can involve specific tasks, recording conditions, diagnostic criteria, or populations, that can limit the utility of combining different datasets (65, 70, 87). Some conditions are also rarer, which can make recruitment difficult and time-consuming for researchers (65). Some datasets also lack sensitive characterizing information about participant, like some demographic information or comorbidities, making it difficult to use for research groups (93).

#### 3.2.2 Modality-specific implications

##### 3.2.2.1 Privacy and security

Patient privacy and confidentiality are major concerns for any type of clinical data collection or clinical monitoring approach, but the engineering and computing behind privacy-preserving techniques for voice and speech data present unique difficulties. Encryption, anonymization, and de-identification, typical safeguards that assure the privacy of people, have proven to be more challenging with voice and speech data than with other biometric information (like facial or iris recognition) (73, 81, 88, 89, 94–96). This is because techniques cannot readily be transferred (the variability in speech signals means that speakers don't utilize a template or print in the same way as other biometric data) (74), or are otherwise limited in their effectiveness (97, 98). Those considerations also extend to third-party individuals if their voice and speech are also being recorded by sensors (73). And while specific privacy-preserving techniques and methodologies are mentioned (for example, automatic feature extraction, subsampling (75), federated learning (65), encryption of voice and speech data (27, 75), and privacy-by design (71), those can quickly become obsolete as new technologies emerge to intrude and access data in an unauthorized manner.

There is also evidence that information about a speaker, especially sociodemographic attributes, can be gathered from a voice recording alone (27, 74, 91, 98). This can include (but is not limited to) body measurements, age, gender, sleepiness, level of intoxication, native language, and socioeconomic status (91), as well as sexual preferences (71), some of which correspond to personal identifiable information (PII) (98). But, up to now, the discourse surrounding privacy in voice and speech data has paid little attention voice-inferred information (91) and how its understanding can reinforce concerns surrounding data privacy and second-hand analysis of data (67). However, it is important to mention that the collection of voice and speech data can mean a lot of different things. As such, different types of voice and speech data (speech, voice, breathing, laughing, coughing, etc.) present different risks (76, 98). For example, breathing sounds generally contain less sensitive information than speech and audio recordings of conversations (99).

Finally, one article mentions the risk of the recording of unflattering or nefarious content (for example recording of people in the bathroom, but also more deeming content like recordings of threats or other illegal activities), specifically through continuous voice recording (100). This could entail the possibility that research groups must break confidentiality for legal reasons (100). Another article also mentions that the privacy of conversational home devices, which can be used to capture voice and speech data, is not guaranteed, as it has already been shown that private conversations have been sent to unintended recipients (83).

##### 3.2.2.2 Informed consent

Ensuring informed consent prior to the data collection of voice and speech is essential. As mentioned in several articles, one of the major advantages of voice and speech biomarkers is the ability to analyze data continuously instead of only during the occasional, punctual encounters with their healthcare professionals (84, 97, 98, 101, 102). But continuous recording, in addition to coming with issues of risk to individual privacy, may also imply the recording of people who are not aware that they are being recorded and, perhaps most importantly, have not consented to that recording (68, 73, 76, 79, 88, 94, 95, 100). This can include people in the background who are just passing within range of the recording device or people having a conversation with patients (79). Gathering consent from those individuals is, however, highly unfeasible. Not only can it place patients in uncomfortable situations where they might have to disclose their conditions to people they would not normally divulge it to (76, 92), but it is also far too demanding.

Some articles also highlight the fact that it may be difficult to consent to continuous recording because people cannot know in advance the events that they will encounter and will want to keep private (75, 76). Also, the large amount of voice and speech data gathered increases the privacy risks for people (70). And, since different types of data can be collected with a voice recording (e.g., breathing, free speech), voice and speech data can present somewhat of a unique challenge compared to other types of data collection (100).

Finally, as with other types of data, there is a risk of uncovering incidental finding related to a person's health (70). This could happen, for example, if research is conducted on a sample from an asymptomatic person (75). However, even if the norm is to allow people to decide, at the time of consent, if they would like to be informed of incidental findings (70), and to share the diagnosis, especially if early treatment is available and beneficial (75, 94, 103, 104), sharing results from voice analysis can be risky, as clinical utility of voice biomarker results is uncertain (75).

##### 3.2.2.3 Data

###### 3.2.2.3.1 Quality of Data

The quality of the speech and voice data used to train algorithms and analyze the voice and speech of patients is a key ethical concern in this field. The type of device used, the environment, and other people in the recordings are all different real-world issues that can hinder the effectiveness of the analysis of voice and speech biomarkers (69, 105–108). One paper also mentions that the use of mobile devices in research studies is practical (mimics natural behaviors, less expensive than providing specific study devices), but that it can lead to noise from differences in hardware and software that may need to be corrected before the data can be analyzed and may hinder sharing across research groups (69). Additionally, there are no standardized data formats and recording protocols for voice and speech recordings in healthcare, making it difficult to develop large databases from different sources (27, 65). This poses a problem given the need for a sufficiently large quantity of data to be able to train AI and ML algorithm (75).

Finally, because voice and speech can be affected by energy levels, the timing of voice data collection is crucial. Information about the patients energy levels should thus be noted (81).

###### 3.2.2.3.2 Quantity of Data

Several articles mention that the limited number of substantial, clinically relevant voice datasets is an issue to train algorithms (65, 75, 107–110). However, collecting more data is not necessarily a viable solution, as storage of a large quantity of data can be expensive and require substantial equipment for individual researchers or teams (82, 90, 108, 109, 111). The storage capacities of research groups can also directly impact groups' data collection quantity and preferences (70). In addition, data collection is time intensive for research teams, especially if data needs to be edited to remove unwanted audio and transferred to another format (111). Kröger & al. mention that there is an imbalance in the availability of voice and speech data between private companies, whose data collection is often “technically authorized but not actively consented” (82), and publicly funded research teams, which rely on available datasets or who have to gather data themselves (91).

Several articles identified the small number of training data as one of the limitations of their studies or of other studies they reviewed (87, 93, 101, 105, 107). One author also mentions that some studies even publish without the presence of a control group (101). There is also a lack of longitudinal data on voice and speech that include data from acute illness and beyond (93). The lack of availability of large-scale studies means that the switch from feasibility studies to large-scale development of voice biomarkers will be hard (93).

###### 3.2.2.3.3 Validation of Data

There are concerns about validating the results of voice and speech analysis, raising questions about the clinical introduction of technologies based on voice and speech biomarkers. The ability to validate results from multiple populations, in multiple settings, and with a good enough reliability will be key to introducing the use of voice and speech biomarkers into the clinical care (70, 75, 91, 94, 109, 111, 112). Moreover, some authors question the validity of voice and speech analysis that is detached from the person, mentioning the false interpretations that can happen once the voice and speech data are taken out of context or analyzed without some critical background information on that person (93, 113). Finally, various studies mention the need to validate the results from vocal and speech biomarkers with “gold standards” (27) or other biomarkers (27, 76, 77, 79, 94, 109, 114, 115). Without such standards, the switch from feasibility studies to large-scale development of voice biomarkers will not happen (27). Proper validation of diverse samples and datasets will be essential to scale-up voice and speech biomarker analysis (27, 65, 69, 87, 111, 114). This will mean validating results in different conditions (72, 79), but also for different populations and sub-populations (69, 108).

##### 3.2.2.4 Respect for People

Voice and speech data collection can demand that people perform certain tasks like breathing, reading texts, talking about a subject for a couple minutes, etc. (82). For some conditions targeted by research on voice and speech biomarkers, those tasks can prove to be difficult. For example, Petti et al. (75) mention that for individuals with Alzheimer's disease, some tasks can be humiliating and can cause distress, resulting in frustration and anger (75).

## 3.3 Social implications

### 3.3.1 Conventional implications

#### 3.3.1.1 Bias and lack of diversity

Bias in datasets can contribute to unfair, unbalanced, and even discriminatory studies and analysis of voice and speech biomarkers (27, 65, 68, 72, 76, 83, 85, 98, 100, 102, 107, 114). Biased datasets may not adequately represent the diversity of human emotions across cultures, genders, and age groups (88, 103, 116). Given that an AI or ML algorithms are susceptible to “learning” biases present in the training data, it is imperative to tackle these considerations early on in their development, since all the previous mentioned implications can be perpetuated once an algorithm is integrated into clinical care (6, 46, 65, 82, 85, 86, 89, 93, 100, 108, 116, 117).

Bias mitigation, both in the training datasets and in the validation datasets is essential (27, 46, 82, 83, 85, 88, 92, 94, 103, 104, 107, 116, 118–121). This type of mitigation will require the involvement of diverse stakeholders to be able to understand and consider their different needs and contexts (82, 118).

#### 3.3.1.2 Discrimination

One paper in this review also discussed how the use of “big data” in healthcare could enable discrimination and predatory practices. While this is not specific to voice and speech, they do mention how this could disproportionately affect some individuals with mental health conditions, one category of condition that is extensively researched in the voice biomarkers field (72). The accumulation of emotional data about a person could lead to unintended consequences, like emotional profiling, which could also lead to discrimination (68).

### 3.3.2 Modality-specific implications

#### 3.3.2.1 Bias and lack of diversity

Although the analysis of voice and speech biomarkers using AI is potentially a way to capture a noninvasive (69, 75, 77, 93, 98, 99), relatively inexpensive (93, 99, 101, 106) snapshot of cognition, tissue integrity, and motor function, issues arise related to bias and representation in datasets and algorithms. “Typical adult speakers” (i.e., people without pathological speech, adults, people not in minority communities) appear to be overrepresented in databases (92), and gender and racial disparities remain an unexplored area in voice and speech research and technology development (46). Additionally, when research projects look specifically at gender representation in data, they find that men are disproportionately represented (86). Additionally, to be used on a large scale, voice and speech data must be linked with sociodemographic information about a person, which could increase pre-existing types of discrimination (27).

Increasing the size (89) and diversity of datasets is often one of the suggested solutions to account for biases in datasets (27, 122). However, doing so for voice and speech is a challenge as diversity in voice and speech is difficult to determine and measure. Diversity can be defined demographically, referring to gender, age, sex, ethnic origin, place of birth, and/or first language (102, 107). Alternatively, it can be defined by the type of disease or condition, or by the stage in their development. Regarding speech and voice, diversity can also refer to the range of languages, dialects, and accents or the type of data (free speech, written text read, breath work, common sounds, etc.). Even within one language, there can be some linguistic variation, or “orderly hierarchy,” that can be critical to understanding one's speech. And while speech datasets usually specify languages spoken, more granularity in linguistic categorization is needed (107). There is also diversity in the type of recording device (professional microphone vs. phone microphone vs. ambient recording, presence of background noise, etc.), and this technical diversity can directly impact the validity and quality of analysis (107). However, because voice and speech are often related to language, detection of native language can represent personal information that has the potential to be misused to detect and discriminate against minorities (91). Moreover, there also seems to be some misperceived understanding that voice and speech samples are an unbiased form of data (116).

#### 3.3.2.2 Discrimination

Although discrimination in data collection is an ongoing consideration and topic of research, Anglade & al (118). specifically mentions that people with communication disorders like aphasia, a condition that could benefit greatly from the use of voice and speech biomarkers, are often excluded from research because of the methodological or ethical challenges or adaptations that can come with including those communities in research (118). This consideration can also include individuals with cognitive impairment or people with acute psychiatric conditions (96). Finally, and most importantly, Dikaios et al. (93) mention that few studies on speech analysis acknowledge sources of bias and show any attempt to control them, hinting that much work needs to be done to assess and address those issues.

#### 3.3.2.3 Exploitation and manipulation

One article also delve deeper into the dangers of voice and speech data access and sharing, especially regarding data breaches (94). Voice forgery and doxing is of particular concern in recent times, but it is not out of the realm of possibility that recordings could be used in prosecution or even divorce proceedings (92).

### 3.4 Legal implications

#### 3.4.1 Conventional implications

There are a number of legal implications surrounding voice and speech biomarker data that are consistent with those reported in the literature about other types of health data. Articles also mention how the use of voice biomarkers could lead to some legal implications.

##### 3.4.1.1 Compliance across multiple jurisdictions

Legal implications can arise from the laws and regulations in place where voice and speech data is collected and where it is stored, and these are thus important for researchers to consider when planning and implementing research projects. This process is sometimes complicated when local regulations from the city where the participant is residing differ from the those of the province, state, or even country, or when participants move from one place to another with the device that captures their voice and speech. Furthermore, local, state, and national regulations regarding the collection and storage or data can conflict. For example, Casillas & Cristia (88) give out the example of a local regulation that would ban the recording of data in a specific environment (like supermarkets or shops), and where state or national regulations consider the recording of this type of data in this environment as public (88). Some concerns have also been raised around the ownership of the data gathered in research, and if it belongs to the researchers or the participants in research (81).

Another way to mitigate bias and ensure diversity is to facilitate the sharing of voice and speech data between researchers (27). However, due to legislative and regulatory discrepancies surrounding voice and speech data and its dissemination, data sharing between research teams is difficult. Developing policies that facilitate the *safe* sharing of voice and speech data is of utmost importance to ensure robust AI and ML analysis of voice and speech biomarkers (82, 87).

##### 3.4.1.2 Safeguarding participants in data-driven trials

Additionally, one article highlighted concerns about the regulatory implications for research participants, particularly in cases where private companies conduct clinical trials using digital samples (like voice and speech) rather than biological ones. Unlike university-based research teams that receive federal funding and must comply with the Common Rule, these private companies are not necessarily subject to FDA oversight, the Common Rule, or other federal regulations (120). The article further explores this issue, noting that there appears to be a general consensus within the research ethics community that extending the Common Rule to all human subjects research would be both impractical and difficult to enforce (120).

##### 3.4.1.3 Data ownership

Data ownership is a common consideration in research and clinical care involving data collection or the use of personal data. However, for speech and voice data, it seems particularly important for Indigenous communities, where cultures and traditions are often carried out orally. In that sense, voice and speech can be related to people's rights and ownership over their cultural heritage, and merit special consideration (123). Finally, Woodward & al. mention that well-being apps lack in basic privacy policies and can sell their user's information to data brokers and that international regulations have failed in their attempts to give control to citizens over their personal data (121).

#### 3.4.2 Modality-specific implications

A vast majority of articles address how devices and teams that gather, store, and share voice data need to follow international and national regulatory frameworks. However, one article mentioned that it can be difficult to determine if the data falls under certain privacy laws (97). Also, as mentioned previously, there is no universal definition of privacy, and no clear understanding of when regulations apply to the capture, storage, and processing of speech data (71). This also extends to a lack of common understanding and different interpretations of laws and regulations regarding speech data and speech technologies between legal and technical communities (71). For authors, privacy cannot be ensured without it having a universal definition and legal provisions of it as an enforceable right (71).

Using voice and speech as biomarkers leads to issues that straddle those of traditional biomarkers and challenges regarding digital health (27, 91). Some questions have also been raised about the possibilities of allowing higher forms of data protection to some information that can be determined with the use of voice biomarkers, such as an individual's emotions and behavioral traits (76). There are also issues surrounding the privacy of voice data in civil proceedings (like divorce and litigation) or criminal cases, and questions about if private information could be subpoenaed (70).

## 4 Discussion

This review examined the ethical, legal, and social implications (ELSI) of voice and speech data in healthcare, focusing on whether their use presents novel or exceptional challenges. Many of the identified ELSI are not unique to voice and speech data. These concerns have been extensively discussed in the context of biomedical technologies and other types of biomarkers (124–127). However, the distinctive features of voice and speech require reinterpreting existing frameworks.

Tables 2–4 present a side-by-side to illustrate how the use of voice and speech data in healthcare both aligns with conventional implications or brings out modality-specific implications. Ethical concerns such as privacy, consent, and data quality persist, but take on new dimensions due to the technical and inferential characteristics of voice. Social implications reveal how structural biases are amplified through underrepresentation and linguistic exclusion, while also introducing risks of exploitation specific to audio data. Legally, although some regulatory gaps are familiar, voice data introduces new complications in classification, protection, and admissibility. The divergence in these columns shows where existing frameworks are strained and where entirely new considerations arise. This type of analysis is important because the unique characteristics of voice introduce challenges not captured by existing discussions of other health data. Integrating these concerns into broader ELSI conversations ensures that the distinct risks and responsibilities tied to voice are recognized, while maintaining coherence across debates about health data governance.

**Table 2:** Ethical implications—conventional vs. modality-specific implications.

Ethical Implication	Conventional implications	Modality-specific implications
Privacy and Security	– Risks from continuous, passive collection of voice data. Storage and sharing of data in repositories raises risks of breaches, leaks, and re-identification – Voice and speech recordings can be perceived as surveillance or intrusiveness by patients/caregivers. Potential for population-level surveillance. – Tension between data quantity vs. privacy.	– Risks from technical limits of privacy-preserving methods (encryption, anonymization, de-identification). Methods are also quickly outdated as new intrusion tech emerges. – Possible to extract voice-inferred information from speech and voice recordings (age, gender, socioeconomic status) – Privacy and security considerations can extends to third-party voices (bystanders) and different types of voice data (speech, breathing, laughing, etc.) with varying sensitivity.
Informed Consent	– Difficulty ensuring participants understand risks of data leakage and long-term harms. – General and technical literacy affects how participants interpret consent forms; need for accessible language.	– Challenges in obtaining consent for continuous recording, including risks to non-consenting third parties. – Practical impossibility of gathering consent from all affected individuals, and for future events participants may want to keep private.
Data Implications	– Difficulties in sharing datasets between research groups due to format, task-specificity, and population differences. – Data stored in inconvenient or incompatible formats; heterogeneity in recording conditions and criteria that can limit the ability to combine or re-use datasets across groups. – Recruitment is difficult, especially for rare conditions; datasets often lack key demographic/comorbidity information.	– Ensuring quality, standardization, and validation of voice/speech data for algorithm training and clinical application. – Lack of standardized formats and protocols; device and environment variability; mobile devices add hardware/software noise. – Concerns about false interpretations when data analyzed out of context; need to control for energy levels, timing, and recording conditions.
Respect for People		– Some recordings tasks can be difficult or limited due to condition, energy levels.

**Table 3:** Social implications—conventional vs. modality-specific implications.

Social Implication	Conventional implications	Modality-specific implications
Bias and Diversity	– Bias framed as a technical and social problem that must be addressed in development pipelines. – Bias in datasets and algorithms risks leading to unfair, unbalanced, or discriminatory outcomes in clinical care.	– Voice/speech samples are often wrongly perceived as unbiased, which hides structural inequities. – Bias in voice data not only skews results but also risks misuse for discrimination against minorities (e.g., via language detection). – Overrepresentation of “typical adult speakers” and men; underrepresentation of minorities, children, and pathological speech.
Discrimination	– Risks of discrimination and predatory practices from large-scale health data use, especially for individuals with mental health conditions.	– Exclusion of vulnerable groups (e.g., communication disorders, cognitive impairment, acute psychiatric conditions) from research.
Exploitation		– Risks associated with the nefarious use of voice and speech data in case of data leakage (doxing, misrepresentation of people), or use of voice and speech data in legal proceedings.

**Table 4:** Legal implications—conventional vs. modality-specific implications.

Legal Implication	Conventional implications	Modality-specific implications
Regulatory Complexity	– Compliance with existing legal frameworks, emphasizing the need to account for overlapping and sometimes conflicting regulations across local, state, national, and international levels, especially when it comes to the sharing of voice and speech data.	– Highlights the conceptual ambiguity of implications, such as privacy itself, stressing the absence of a universal definition and enforceable right, which limits the applicability of existing laws, as well as divergent interpretation between legal and technical communities.
Data Ownership	– Extends ownership debates to voice as cultural heritage, particularly for Indigenous communities, linking privacy and ownership to collective rights and traditions.	
Other Use of Data	– Gaps in privacy policies of well-being apps and risks of user data being sold to third parties, emphasizing the need for compliance with existing regulations.	– Raises broader questions about whether voice data should be protected from legal compulsion and how privacy rights extend into judicial contexts.

A key observation is the interdependence of ethical, legal, and social implications in this domain. For example, ensuring diversity in voice datasets is more complex than in other biomarker domains (27). Although diversity is essential for reducing bias, building representative voice datasets is resource-intensive and logistically difficult. This challenge is compounded by fragmented legal protections and inconsistent privacy regulations. Several articles question whether existing national and international regulations adequately govern voice biomarker development. While GDPR and HIPAA provide some safeguards to patients and system users (72, 88, 97, 116, 121), their limitations are evident, especially since many companies rely on them as default governance models (109). National and international regulatory frameworks must be adapted to address the specific risks posed by new biomarkers, including voice-based digital biomarkers (71, 78). Regulatory responses must keep pace with rapid industry development and clarify legal responsibility (71, 91). Beneath these regulatory challenges lie unresolved technical uncertainties. While outside the scope of this review, these gaps limit a comprehensive understanding. Nevertheless, the recurring mention of technical issues (such as inconsistent labeling and processing) shows their influence on both ELSI and mitigation strategies.

Addressing the intersection of ethical, legal, social, and technical implications requires transdisciplinary and cross-sector engagement. As shown, the reviewed literature spans voice technology, machine learning, AI, and digital health instrumentation. Development of AI tools must incorporate insights from adjacent disciplines and from stakeholders (e.g., patients, clinicians, and advocates) throughout the design pipeline and medical AI lifecycle (50, 71, 91). The field of bioethics can offer guidance in curating these discussions, ensuring stakeholder representation and participation, and generating context-specific guidelines that go beyond overarching principles and general claims about privacy, informed consent, and data security (50).

Few articles included in this review addressed the private sector, likely due to the methodological constraints of the search strategy and inclusion criteria. Those that did, however, tended to highlight the widening gap between academic researchers and private industry (82, 91). This was mostly due, as reported in the results, to the scarcity of large, diverse, high-quality datasets (36, 37, 40, 128, 129). This limitation motivated initiatives like the Bridge2AI-Voice consortium (130), which aims to develop an ethically sourced database of thousands of voice samples linked to health metadata (34, 49, 131). Historically, large-scale data collection projects from healthcare institutions have contributed to advance considerations for the responsible stewardship and governance of data (132, 133), as well as providing viable solutions for the lack or inaccessibility of relevant data for research teams (134). But, as shown by (91), some researchers seek access to non-clinical voice recordings, such as those held by tech companies or in public repositories. These datasets, while abundant, often lack proper curation and metadata annotation needed for healthcare applications (135, 136), something that appears to be lacking in private datasets (132). Collaboration between academic and industry sectors remains valuable, especially as private actors lead technological development in this domain (137). Yet, persistent communication gaps and contradictory incentive schemes across sectors impede alignment and slow field advancement (67).

The literature remains sparse on ELSI specific to voice and speech data in healthcare. Though the field is relatively new, having emerged in the early 2000s (138), recent years show a growing engagement with ethical, legal, and social dimensions. Despite this trend, empirical studies and in-depth conceptual analyses are still lacking. Most reviews mention ELSI superficially without probing underlying assumptions or stakeholder perspectives. This gap underscores the need for further research, particularly qualitative work exploring contributors' views on privacy, consent, data usage, and trust. Understanding the motivations and concerns of data subjects is critical for ethical implementation.

Most publications on voice and speech data in healthcare originate from researchers based in the Global North (see Figure 3). This reflects the geographic concentration of datasets, infrastructure, and funding, as well as restrictive international privacy regulations that complicate cross-border data sharing (71, 88, 97). These conditions limit international collaboration and prevent research teams in low- and middle-income countries (LMICs) from accessing or contributing to existing datasets. The same imbalance holds for industry: most companies developing voice AI tools are based in the West (50). While this geographic clustering may ease local industry–academia collaboration, it undermines global inclusivity. LMICs often lack the technical, financial, and institutional capacity to generate or refine voice data (132, 139). This exclusion means that a large portion of the global population is left out of both the development and the benefits of voice biomarker technologies. Moreover, research priorities and disease targets are shaped by Global North interests. The COVID-19 pandemic exemplifies this dynamic: while voice AI tools for cough analysis showed promise, their global utility was undermined by training data biases skewed toward wealthier regions (140, 141). This case underscores how big data production mirrors (and reinforces) existing global inequities (142, 143).

As mentioned, one of the central questions in this review is whether the use of voice and speech data in healthcare constitutes a form of exceptionalism. Rather than endorsing voice biomarker exceptionalism (i.e., a claim that this domain requires entirely new ethical frameworks), this paper argues for *contextualism*. Contextualism, as drawn from bioethical debates in genomics and neuroethics, rejects binary categories (exceptional vs. non-exceptional) and instead emphasizes that ethical implications vary according to the specific features of the data, its use context, and the sociotechnical systems surrounding it (144–147). Voice data, in this framing, is neither wholly new nor entirely analogous to prior biomarker domains. It presents a “fundamental duality” (145), carrying specific risks (e.g., such as the inferential excesses of emotional AI, the misclassification of linguistic minorities, or the covert extraction of demographic attributes), but these risks are best addressed by adapting existing frameworks to new contexts, rather than discarding them. The ELSI mentioned in this review are not necessarily unique to voice and speech biomarkers, but they do take on different meanings and understandings and require new mitigation solutions given the unique characteristics of these biomarkers. The question is, however, interesting and even important to ask.

These contributions support a *contextualist turn*: ethical frameworks should not presume novelty but must remain responsive to the specific risks and affordances of voice AI. In this light, the development of voice and speech biomarkers should be understood as part of a broader evolution in digital health, comparable to past transitions seen in genomics or neuroimaging (148–151). Lessons from those domains (about oversight gaps, translational bottlenecks, and stakeholder exclusion) are not just relevant, but necessary guides for the governance of voice data in health.

## 5 Limitations

This review has several limitations that reflect both structural constraints in the field and methodological boundaries inherent to scoping reviews. First, although this review refers to “voice and speech data” as a combined category, the technical distinction between voice and speech remains imprecise in the reviewed literature. Voice generally refers to the acoustic signal produced by the vocal tract, while speech encompasses the linguistic content of that signal. These modalities are often treated interchangeably, but raise distinct ethical, legal, and social concerns depending on which is being collected or analyzed. This lack of definitional consensus limits the granularity with which ELSI concerns can be differentiated and analyzed (43). Second, private industry perspectives are significantly underrepresented in the reviewed material. While it is common for industry work to remain unpublished or appear in grey literature, the academic databases consulted (e.g., MEDLINE, Web of Science, EMBASE, IEEE) primarily index peer-reviewed sources. As a result, commercial practices and proprietary standards (especially around data acquisition, model training, and consent) are largely absent from this analysis. This is only compelled by the current state of voice AI health-tech sector, which currently offers limited disclosure and transparency on their public-facing websites about their policies (152) and of their technology (153). Third, while many included articles mention ELSI, their engagement is often brief or speculative rather than empirical or conceptually grounded. This limits the capacity of the review to draw out structured, well-theorized distinctions across domains, particularly in areas such as incidental findings, third-party data capture, or intergenerational data ethics. Fifth, as a scoping review, this article maps the breadth of existing literature rather than evaluating its quality or methodological robustness. This is appropriate for identifying gaps and setting research agendas but limits the evidentiary strength for normative claims or policy recommendations.

## 6 Conclusion

To our knowledge, this is the first scoping review examining the ELSI associated with the collection and use of voice and speech data in healthcare. Reviewing literature is an important initial step in conducting ethical foresight. It allows to identify issues and implications that are under consideration, uncover those which are not yet acknowledged, and highlight those for which further attention is required. The emergence of voice and speech-based biomarkers reframes how voice is understood; not only as expression, but as a data source conveying cognitive, physiological, and affective information. Voice and speech have long served as instruments of identity, expression, and social interaction. They are now increasingly central not only to human communication, but also to human-machine interaction. Voice also operates as a metaphor for representation, agency, and political visibility (154). With the advent of voice biomarkers, voice is no longer just symbolic, it is also a computational object for encoded cognitive, neuromuscular, and physiological signals (155, 156).

This review also identifies several areas where additional research and policy development are urgently needed. In particular, the collection and use of voice and speech data in health contexts raise distinctive ethical concerns due to their capacity to reveal sensitive, inferential health information. The future trajectory of voice biomarker research remains uncertain, especially given the difficulty of building datasets that are trustworthy, clinically meaningful, and shareable across contexts. This review aims to support the ethical development, deployment, and governance of voice and speech data practices in healthcare and related domains.

## Footnotes

^1^Voice, or vocalization, is the sound made by the vibration between vocal folds in the larynx when air is pushed by the lungs (2). Speech, on the other hand, refers to the human vocalization made by the coordinated muscle movement of the lips and tongue that shapes recognizable sounds (5). Speech also involves prosody, lexicality and linguistic features like spoken words that can also contain potential biomarkers of diseases (6). However, for easier comprehension, this article will refer to voice and speech as one and the same.

## Group members of the Bridge2AI-Voice consortium

University of South Florida, Tampa, FL, US: Yael Bensoussan. Weill Cornell Medicine, New York, NY, USA: Olivier Elemento. Weill Cornell Medicine, New York, NY, USA: Anais Rameau. Weill Cornell Medicine, New York, NY, USA: Alexandros Sigaras. Massachusetts Institute of Technology, Boston, MA, USA: Satrajit Ghosh. Vanderbilt University Medical Center, Nashville, TN, USA: Maria Powell. University of Montreal, Montreal, Quebec, Canada: Vardit Ravitsky. Simon Fraser University, Burnaby, BC, Canada: Jean Christophe Belisle-Pipon. Oregon Health & Science University, Portland, OR, USA: David Dorr. Washington University in St. Louis, St. Louis, MO, USA: Phillip Payne. University of Toronto, Toronto, Ontario, Canada: Alistair Johnson. University of South Florida, Tampa, FL, USA: Ruth Bahr. University of Florida, Gainesville, FL, USA: Donald Bolser. Dalhousie University, Toronto, ON, Canada: Frank Rudzicz. Mount Sinai Hospital, Sinai Health, University of Toronto, Toronto, ON, Canada: Jordan Lerner-Ellis. Boston Children’s Hospital, Boston, MA, USA: Kathy Jenkins. University of Central Florida, Orlando, FL, USA: Shaheen Awan. University of South Florida, Tampa, FL, USA: Micah Boyer. Oregon Health & Science University, Portland, OR, USA: William Hersh. Washington University in St. Louis, St. Louis, MO, USA: Andrea Krussel. Oregon Health & Science University, Portland, OR, USA: Steven Bedrick. UT Health, Houston, TX, USA: Toufeeq Ahmed Syed. University of South Florida, Tampa, FL, USA: Jamie Toghranegar. University of South Florida, Tampa, FL, USA: James Anibal. New York, NY, USA: Duncan Sutherland. University of South Florida, Tampa, FL, USA: Enrique Diaz-Ocampo. University of South Florida, Tampa, FL, USA: Elizabeth Silberhoz Boston Children’s Hospital, Boston, MA, USA: John Costello. Vanderbilt University Medical Center, Nashville, TN, USA: Alexander Gelbard. Vanderbilt University Medical Center, Nashville, TN, USA: Kimberly Vinson. University of South Florida, Tampa, FL, USA: Tempestt Neal. Mount Sinai Health, Toronto, ON, Canada: Lochana Jayachandran. The Hospital for Sick Children, Toronto, ON, Canada: Evan Ng. Mount Sinai Health, Toronto, ON, Canada: Selina Casalino. University of South Florida, Tampa, FL, USA: Yassmeen Abdel-Aty. University of South Florida, Tampa, FL, USA: Karim Hanna. University of South Florida, Tampa, FL, USA: Theresa Zesiewicz. Florida Atlantic University, Boca Raton, FL, USA: Elijah Moothedan. University of South Florida, Tampa, FL, USA: Emily Evangelista. Vanderbilt University Medical Center, Nashville, TN, USA: Samantha Salvi Cruz. Weill Cornell Medicine, New York, NY, USA: Robin Zhao. University of South Florida, Tampa, FL, USA: Mohamed Ebraheem. University of South Florida, Tampa, FL, USA: Karlee Newberry. University of South Florida, Tampa, FL, USA: Iris De Santiago. University of South Florida, Tampa, FL, USA: Ellie Eiseman. University of South Florida, Tampa, FL, USA: JM Rahman. Boston Children’s Hospital, Boston, MA, USA: Stacy Jo. Hospital for Sick Children, Toronto, ON, Canada: Anna Goldenberg.

## References

[1] Simonyan K Ackermann H Chang EF Greenlee JD. New developments in understanding the complexity of human speech production. J Neurosci. (2016) 36(45):11440–8. 10.1523/JNEUROSCI.2424-16.2016 27911747 PMC5125211

[2] Zhang Z. Mechanics of human voice production and control. J Acoust Soc Am. (2016) 140(4):2614. 10.1121/1.4964509 27794319 PMC5412481

[3] Casper JK Leonard R. Understanding Voice Problems: A Physiological Perspective for Diagnosis and Treatment. Philadelphia: Lippincott Williams & Wilkins (2006). p. 528.

[4] Kent RD. The uniqueness of speech among motor systems. Clin Linguist Phon. (2004) 18(6–8):495–505. 10.1080/02699200410001703600 15573486

[5] Lin H Karjadi C Ang TFA Prajakta J McManus C Alhanai TW Identification of digital voice biomarkers for cognitive health. Explor Med. (2020) 1:406–17. 10.37349/emed.2020.00028 33665648 PMC7929495

[6] Schuller B. Voice and speech analysis in search of states and traits. In: Salah AA Gevers T, editors. Computer Analysis of Human Behavior. London: Springer (2011). p. 227–53. Available online at: 10.1007/978-0-85729-994-9_9 (Accessed October 24, 2024).

[7] Kamiloğlu RG Sauter DA. Voice production and perception. Oxf Res Encycl Psychol. (2021). 10.1093/acrefore/9780190236557.013.766 Available online at: https://oxfordre.com/psychology/display/10.1093/acrefore/9780190236557.001.0001/acrefore-9780190236557-e-766 (Accessed August 29, 2024)

[8] Pennock Speck B. Voice and the construction of identity and meaning. In: Navarro I Crespo NA, editors. In-roads of Language: Essays in English Studies. Castellón: Publicacions de la Universitat Jaume I (2006). p. 91–102.

[9] Carding P Mathieson L. Voice and speech production. In: Watkinson J Clarke R, editors. Scott-Brown’s Otorhinolaryngology and Head and Neck Surgery. 8th ed. Boca Raton, FL: CRC Press (2018). p. 905–10.

[10] Redford MA. Introduction. In: Redford MA, editor. The Handbook of Speech Production. Hoboken: John Wiley & Sons, Ltd (2015). p. 1–10. Available online at: https://onlinelibrary.wiley.com/doi/abs/10.1002/9781118584156.ch1 (Accessed May 12, 2024).

[11] Schlichter A Eidsheim NS. Introduction: voice matters. Postmod Cult. (2014) 24(3). Available online at: https://muse.jhu.edu/pub/1/article/589565 (Accessed May 12, 2024)

[12] Popov D. Speech personality, individuality and uniqueness of human voice. In: Proceedings of Speech and Language 2019, 7th International Conference on Fundmental and Applied Aspects of Speech and Language. Belgrade: Life Activities Advancement Center (2019). p. 34.

[13] González-Rodríguez J Toledano DT Ortega-García J. Voice biometrics. In: Jain AK Flynn P Ross AA, editors. Handbook of Biometrics. 1st ed. New York, NY: Springer US (2008). p. 151–70.

[14] Boles A Rad P. Voice biometrics: deep learning-based voiceprint authentication system. 2017 12th System of Systems Engineering Conference (SoSE) (2017). p. 1–6 Available online at: https://ieeexplore.ieee.org/abstract/document/7994971 (Accessed July 3, 2024).

[15] Tandogan SE Senear HT Tavli B. Towards measuring uniqueness of human voice. 2017 IEEE Workshop on Information Forensics and Security (WIFS) (2017). p. 1–6. Available online at: https://ieeexplore.ieee.org/abstract/document/8267666?casa_token=F5APTFe2TY4AAAAA:HP8XkJ1fWaAHb2iDRt18NU5L0d9oQBM2qjjyplormlUHs9hG-Cq298K2CY2o8yw6BLZG-SVejpA (Accessed October 24, 2024).

[16] Dahia G Jesus L Segundo MP. Continuous authentication using biometrics: an advanced review. Wiley Interdiscip Rev Data Min Knowl Discov. (2020) 10(4):e1365. 10.1002/widm.1365

[17] Li H Xu C Rathore AS Li Z Zhang H Song C Vocalprint: exploring a resilient and secure voice authentication via mmWave biometric interrogation. In*:* Proceedings of the 18th Conference on Embedded Networked Sensor Systems. New York, NY, USA: Association for Computing Machinery (2020). p. 312–25. (SenSys ‘20). Available online at: https://dl.acm.org/doi/10.1145/3384419.3430779 (Accessed October 24, 2024).

[18] Laput G Ahuja K Goel M Harrison C. Ubicoustics: plug-and-play acoustic activity recognition. In: Proceedings of the 31st Annual ACM Symposium on User Interface Software and Technology. New York, NY, USA: Association for Computing Machinery (2018). p. 213–24. (UIST ‘18). Available online at: https://dl.acm.org/doi/10.1145/3242587.3242609 (Accessed October 24, 2024).

[19] Berisha V Liss JM. Responsible development of clinical speech AI: bridging the gap between clinical research and technology. Npj Digit Med. (2024) 7(1):1–12. 10.1038/s41746-024-01199-1 39122889 PMC11316053

[20] FDA-NIH Biomarker Working Group. BEST (Biomarkers, EndpointS, and Other Tools) Resource. Silver Spring (MD): Food and Drug Administration (US) (2016). Available online at: http://www.ncbi.nlm.nih.gov/books/NBK326791/ (Accessed October 24, 2024). 27010052

[21] Idrisoglu A Dallora AL Anderberg P Berglund JS. Applied machine learning techniques to diagnose voice-affecting conditions and disorders: systematic literature review. J Med Internet Res. (2023) 25(1):e46105. 10.2196/46105 37467031 PMC10398366

[22] Motahari-Nezhad H Fgaier M Mahdi Abid M Péntek M Gulácsi L Zrubka Z. Digital biomarker–based studies: scoping review of systematic reviews. JMIR MHealth UHealth. (2022) 10(10):e35722. 10.2196/35722 36279171 PMC9641516

[23] Powell D. Walk, talk, think, see and feel: harnessing the power of digital biomarkers in healthcare. Npj Digit Med. (2024) 7(1):1–3. 10.1038/s41746-024-01023-w 38396034 PMC10891042

[24] Au R Kolachalama VB Paschalidis IC. Redefining and validating digital biomarkers as fluid, dynamic multi-dimensional digital signal patterns. Front Digit Health. (2021) 3:751629. 10.3389/fdgth.2021.751629 35146485 PMC8822623

[25] Babrak LM Menetski J Rebhan M Nisato G Zinggeler M Brasier N Traditional and digital biomarkers: two worlds apart? Digit Biomark. (2019 ) 3(2):92–102. 10.1159/000502000 32095769 PMC7015353

[26] Mulinari S. Short-circuiting biology: digital phenotypes, digital biomarkers, and shifting gazes in psychiatry. Big Data Soc. (2023) 10(1):20539517221145680. 10.1177/20539517221145680

[27] Fagherazzi G Fischer A Ismael M Despotovic V. Voice for health: the use of vocal biomarkers from research to clinical practice. Digit Biomark. (2021) 5(1):78–88. 10.1159/000515346 34056518 PMC8138221

[28] Pizzimenti M Kalia A Toghranegar JA Ebraheem M Cummings N Ghosh SS Consensus-Based Definitions for Vocal Biomarkers: The International VOCAL Initiative. medRxiv. (2025). p. 2025.10.23.25338518. Available online at: https://www.medrxiv.org/content/10.1101/2025.10.23.25338518v1 (Accessed November 7, 2025).

[29] Robin J Harrison JE Kaufman LD Rudzicz F Simpson W Yancheva M. Evaluation of speech-based digital biomarkers: review and recommendations. Digit Biomark. (2020) 4(3):99–108. 10.1159/000510820 33251474 PMC7670321

[30] Elbéji A Zhang L Higa E Fischer A Despotovic V Nazarov PV Vocal biomarker predicts fatigue in people with COVID-19: results from the prospective Predi-COVID cohort study. BMJ Open. (2022) 12(11):e062463. 10.1136/bmjopen-2022-062463 PMC9684280 36414294

[31] Rosen-Lang Y Zoubi S Cialic R Orenstein T. Using voice biomarkers for frailty classification. GeroScience. (2023) 46:1175–9. 10.1007/s11357-023-00872-9 37480417 PMC10828289

[32] Greenwood T Nunes N. The coming revolution of voice-based digital biomarkers to diagnose and monitor disease. ZS. (2022). Available online at: https://www.zs.com/content/dam/pdfs/The-coming-revolution-of-voice-based-digital-biomarkers.pdf (Accessed November 17, 2025).

[33] Al-Dewik NI Younes SN Essa MM Pathak S Qoronfleh MW. Making biomarkers relevant to healthcare innovation and precision medicine. Processes. (2022) 10(6):1107. 10.3390/pr10061107

[34] Bensoussan Y Elemento O Rameau A. Voice as an AI biomarker of health—introducing audiomics. JAMA Otolaryngol Neck Surg. (2024) 150(4):283–4. 10.1001/jamaoto.2023.4807 38386315

[35] Seyhan AA Carini C. Are innovation and new technologies in precision medicine paving a new era in patients centric care? J Transl Med. (2019) 17(1):114. 10.1186/s12967-019-1864-9 30953518 PMC6451233

[36] Ahmed S Haigh AMF de Jager CA Garrard P. Connected speech as a marker of disease progression in autopsy-proven Alzheimer’s disease. Brain J Neurol. (2013) 136(Pt 12):3727–37. 10.1093/brain/awt269 PMC3859216 24142144

[37] Tracy JM Özkanca Y Atkins DC Hosseini Ghomi R. Investigating voice as a biomarker: deep phenotyping methods for early detection of Parkinson’s disease. J Biomed Inform. (2020) 104:103362. 10.1016/j.jbi.2019.103362 31866434

[38] Briend F David C Silleresi S Malvy J Ferré S Latinus M. Voice acoustics allow classifying autism spectrum disorder with high accuracy. Transl Psychiatry. (2023) 13(1):1–8. 10.1038/s41398-023-02554-8 37422467 PMC10329669

[39] Adams P Rabbi M Rahman T Matthews M Voida A Gay G Towards personal stress informatics: comparing minimally invasive techniques for measuring daily stress in the wild. Proc—pERVASIVEHEALTH 2014 8th Int Conf Pervasive Comput Technol Healthc (2014). p. 72–9

[40] Shin D Cho WI Park CHK Rhee SJ Kim MJ Lee H Detection of minor and Major depression through voice as a biomarker using machine learning. J Clin Med. (2021) 10(14):3046. 10.3390/jcm10143046 34300212 PMC8303477

[41] Naseer A Rani M Naz S Razzak MI Imran M Xu G. Refining Parkinson’s neurological disorder identification through deep transfer learning. Neural Comput Appl. (2020) 32(3):839–54. 10.1007/s00521-019-04069-0

[42] Raina R Jha RK. Intelligent and interactive healthcare system (I2HS) using machine learning. IEEE Access. (2022) 10:116402–24. 10.1109/ACCESS.2022.3197878

[43] Kalia A Boyer M Fagherazzi G Bélisle-Pipon JC Bensoussan Y. Master protocols in vocal biomarker development to reduce variability and advance clinical precision: a narrative review. Front Digit Health. (2025) 7:1619183. 10.3389/fdgth.2025.1619183 40657648 PMC12247298

[44] Liu GS Jovanovic N Sung CK Doyle PC. A scoping review of artificial intelligence detection of voice pathology: challenges and opportunities. Otolaryngol Neck Surg. (2024) 171(3):658–66. 10.1002/ohn.809 38738887

[45] Bonastre JF Delgado H Evans N Kinnunen T Lee KA Liu X Benchmarking and challenges in security and privacy for voice biometrics. arXiv. (2021). Available online at: http://arxiv.org/abs/2109.00281 (Accessed October 25, 2024).

[46] Chen X Li Z Setlur S Xu W. Exploring racial and gender disparities in voice biometrics. Sci Rep. (2022) 12(1):3723. 10.1038/s41598-022-06673-y 35260572 PMC8904636

[47] Leschanowsky A Rusti C Quinlan C Pnacek M Gorce L Hutiri W. A data perspective on ethical challenges in voice biometrics research. IEEE Trans Biom Behav Identity Sci. (2024):1.

[48] Wells A Usman AB. Trust and voice biometrics authentication for internet of things. Int J Inf Secur Priv IJISP. (2023) 17(1):1–28.

[49] Bélisle-Pipon JC Powell M English R Malo MF Ravitsky V Bensoussan Y. Stakeholder perspectives on ethical and trustworthy voice AI in health care. Digit Health. (2024) 10:20552076241260407. 10.1177/20552076241260407 39055787 PMC11271113

[50] Andreoletti M Haller L Vayena E Blasimme A. Mapping the ethical landscape of digital biomarkers: a scoping review. PLoS Digit Health. (2024) 3(5):e0000519. 10.1371/journal.pdig.0000519 38753605 PMC11098308

[51] Ford E Milne R Curlewis K. Ethical issues when using digital biomarkers and artificial intelligence for the early detection of dementia. Wiley Interdiscip Rev Data Min Knowl Discov. (2023) 13(3):e1492. 10.1002/widm.1492 38439952 PMC10909482

[52] Subbian V Galvin HK Petersen C Solomonides A. *Ethical, legal, and social Issues (E*LSI) in mental health informatics. Ment Health Inform Enabling Learn Ment Healthc Syst. (2021):479–503.

[53] Wright JM Regele OB Kourtis LC Pszenny SM Sirkar R Kovalchick C Evolution of the digital biomarker ecosystem. Digit Med. (2017) 3(4):154. 10.4103/digm.digm_35_17

[54] Armstrong R Hall BJ Waters DJ, Cochrane Update E. Scoping the scope” of a cochrane review. J Public Health Oxf Engl. (2011) 33(1):147–50. 10.1093/pubmed/fdr015 21345890

[55] Munn Z Peters MDJ Stern C Tufanaru C McArthur A Aromataris E. Systematic review or scoping review? Guidance for authors when choosing between a systematic or scoping review approach. BMC Med Res Methodol. (2018) 18(1):143. 10.1186/s12874-018-0611-x 30453902 PMC6245623

[56] Peters MDJ Godfrey C McInerney P Khalil H Larsen P Marnie C Best practice guidance and reporting items for the development of scoping review protocols. JBI Evid Synth. (2022) 20(4):953. 10.11124/JBIES-21-00242 35102103

[57] Pham MT Rajić A Greig JD Sargeant JM Papadopoulos A McEwen SA. A scoping review of scoping reviews: advancing the approach and enhancing the consistency. Res Synth Methods. (2014) 5(4):371–85. 10.1002/jrsm.1123 26052958 PMC4491356

[58] Veritas Health Innovation. Covidence. (2014). Covidence Systematic Review Software. Available online at: https://www.covidence.org/ (Accessed December 14, 2022).

[59] Hawker S Payne S Kerr C Hardey M Powell J. Appraising the evidence: reviewing disparate data systematically. Qual Health Res. (2002) 12(9):1284–99. 10.1177/1049732302238251 12448672

[60] Lumivero. NVivo (Version 14). (2023). Available online at: www.lumivero.com (Accessed March 27, 2025).

[61] Neale J. Iterative categorization (IC): a systematic technique for analysing qualitative data. Addiction. (2016) 111(6):1096–106. 10.1111/add.13314 26806155 PMC5069594

[62] McGowan J Straus S Moher D Langlois EV O’Brien KK Horsley T Reporting scoping reviews—PRISMA ScR extension. J Clin Epidemiol. (2020) 123:177–9. 10.1016/j.jclinepi.2020.03.016 32229248

[63] Jeong H Jeong YW Park Y Kim K Park J Kang DR. Applications of deep learning methods in digital biomarker research using noninvasive sensing data. Digit Health. (2022) 8:20552076221136642. 10.1177/20552076221136642 36353696 PMC9638529

[64] Ross A Banerjee S Chowdhury A. Deducing health cues from biometric data. Comput Vis Image Underst. (2022) 221:103438. 10.1016/j.cviu.2022.103438

[65] Ganesh S Chithambaram T Krishnan NR Vincent DR Kaliappan J Srinivasan K. Exploring Huntington’s disease diagnosis via artificial intelligence models: a comprehensive review. Diagnostics. (2023) 13(23):3592. 10.3390/diagnostics13233592 38066833 PMC10706174

[66] Genaro Motti V. Towards a design space for emotion recognition. Adjun Proc 2021 ACM Int Jt Conf Pervasive Ubiquitous Comput Proc 2021 ACM Int Symp Wearable Comput (2021). p. 243–7

[67] Miley EV Schaeffler F Beck J Eichner M Jannetts S. Secure account-based data capture with smartphones—preliminary results from a study of articulatory precision in clinical depression. Linguist Vanguard. (2021) 7:20190015. 10.1515/lingvan-2019-0015

[68] Ortiz-Clavijo LF Gallego-Duque CJ David-Diaz JC Ortiz-Zamora AF. Implications of emotion recognition technologies: balancing privacy and public safety. IEEE Technol Soc Mag. (2023) 42(3):69–75. 10.1109/MTS.2023.3306530

[69] Harris C Tang Y Birnbaum E Cherian C Mendhe D Chen MH. Digital neuropsychology beyond computerized cognitive assessment: applications of novel digital technologies. Arch Clin Neuropsychol. (2024) 39(3):290–304. 10.1093/arclin/acae016 38520381 PMC11485276

[70] Carter A Liddle J Hall W Chenery H. Mobile phones in research and treatment: ethical guidelines and future directions. JMIR MHealth UHealth. (2015) 3(4):e4538. 10.2196/mhealth.4538 PMC4704925 26474545

[71] Nautsch A Jasserand C Kindt E Todisco M Trancoso I Evans N. The GDPR & speech data: Reflections of legal and technology communities, first steps towards a common understanding. ArXiv Prepr ArXiv190703458. (2019).

[72] Bauer M Glenn T Monteith S Bauer R Whybrow PC Geddes J. Ethical perspectives on recommending digital technology for patients with mental illness. Int J Bipolar Disord. (2017) 5(1):6. 10.1186/s40345-017-0073-9 28155206 PMC5293713

[73] Lane ND Miluzzo E Lu H Peebles D Choudhury T Campbell AT. A survey of mobile phone sensing. IEEE Commun Mag. (2010) 48(9):140–50. 10.1109/MCOM.2010.5560598

[74] Nautsch A Jiménez A Treiber A Kolberg J Jasserand C Kindt E Preserving privacy in speaker and speech characterisation. Comput Speech Lang. (2019) 58:441–80. 10.1016/j.csl.2019.06.001

[75] Petti U Nyrup R Skopek JM Korhonen A. Ethical considerations in the early detection of Alzheimer’s disease using speech and AI. In: Proceedings of the 2023 ACM Conference on Fairness, Accountability, and Transparency. New York, NY, USA: Association for Computing Machinery (2023). p. 1062–75. (FAccT ‘23). Available online at: https://dl.acm.org/doi/10.1145/3593013.3594063 (Accessed May 16, 2024).

[76] Hurley ME Sonig A Herrington J Storch EA Lázaro-Muñoz G Blumenthal-Barby J Ethical considerations for integrating multimodal computer perception and neurotechnology. Front Hum Neurosci. (2024) 18:1332451. 10.3389/fnhum.2024.1332451 38435745 PMC10904467

[77] Koops S Brederoo SG de Boer JN Nadema FG Voppel AE Sommer IE. Speech as a biomarker for depression. CNS Neurol Disord Drug Targets. (2023) 22(2):152–60. 10.2174/1871527320666211213125847 34961469

[78] Pentland A Lazer D Brewer D Heibeck T. Using reality mining to improve public health and medicine. Stud Health Technol Inform. (2009) 149(ck1, 9214582):93–102. 19745474

[79] Slavich GM Taylor S Picard RW. Stress measurement using speech: recent advancements, validation issues, and ethical and privacy considerations. Stress Int J Biol Stress. (2019) 22(4):408–13. 10.1080/10253890.2019.1584180 PMC7081839 30945584

[80] Bavli I Ho A Mahal R McKeown MJ. Ethical concerns around privacy and data security in AI health monitoring for Parkinson’s disease: insights from patients, family members, and healthcare professionals. AI Soc. (2024) 40:155–65. 10.1007/s00146-023-01843-6

[81] Gold M Amatniek J Carrillo MC Cedarbaum JM Hendrix JA Miller BB Digital technologies as biomarkers, clinical outcomes assessment, and recruitment tools in Alzheimer’s disease clinical trials. Alzheimers Dement Transl Res Clin Interv. (2018) 4:234–42. 10.1016/j.trci.2018.04.003 PMC6021547 29955666

[82] Popp Z Low S Igwe A Rahman MS Kim M Khan R Shifting from active to passive monitoring of Alzheimer disease: the state of the research. J Am Heart Assoc. (2024) 13(2):e031247. 10.1161/JAHA.123.031247 38226518 PMC10926806

[83] Bailey JO Patel B Gurari D. A perspective on building ethical datasets for Children’s conversational agents. Front Artif Intell. (2021) 4:637532. 10.3389/frai.2021.637532 34056578 PMC8155711

[84] Du Y Lubniewski K Price L Breslin G Thomson P Jinadasa N They Can’t believe they’re a tiger”: insights from pediatric speech-language pathologist mobile app users and app designers. Int J Lang Commun Disord. (2023) 58(5):1717–37. 10.1111/1460-6984.12898 37219400

[85] Latif S Ali HS Usama M Rana R Schuller B Qadir J. AI-Based Emotion Recognition: Promise, Peril, and Prescriptions for Prosocial Path. ArXiv Prepr ArXiv221107290. (2022).

[86] Garnerin M Rossato S Besacier L. Gender Representation in Open Source Speech Resources. (2020). p. 6599–605.

[87] Fraser KC Linz N Lindsay H König A. The importance of sharing patient-generated clinical speech and language data. CLPsyc 2019-Sixth Workshop Comput Linguist Clin Psychol. (2019):55–61. 10.18653/v1/W19-3007

[88] Casillas M Cristia A. A step-by-step guide to collecting and analyzing long-format speech environment (LFSE) recordings. Collabra Psychol. (2019) 5(1):24. 10.1525/collabra.209

[89] Low DM Bentley KH Ghosh SS. Automated assessment of psychiatric disorders using speech: a systematic review. Laryngoscope Investig Otolaryngol. (2020) 5(1):96–116. 10.1002/lio2.354 32128436 PMC7042657

[90] Lee A Bessell N van den Heuvel H Saalasti S Klessa K Muller N The latest development of the DELAD project for sharing corpora of speech disorders. Clin Linguist Phon. (2022) 36(2–3):102–10. 10.1080/02699206.2021.1913514 33890543

[91] Kröger JL Lutz OHM Raschke P. Privacy implications of voice and speech analysis–information disclosure by inference. Priv Identity Manag Data Better Living AI Priv 14th IFIP WG 92 96117 116SIG 92 2 Int Summer Sch Wind Switz August 19–23 2019 Revis Sel Pap 14. (2020). 242–58.

[92] Batliner A Hantke S Schuller B. Ethics and good practice in computational paralinguistics. IEEE Trans Affect Comput. (2022) 13(3):1236–53. 10.1109/TAFFC.2020.3021015

[93] Dikaios K Rempel S Dumpala SH Oore S Kiefte M Uher R. Applications of speech analysis in psychiatry. Harv Rev Psychiatry. (2023) 31(1):1–13. 10.1097/HRP.0000000000000356 36608078

[94] Bettis AH Burke TA Nesi J Liu RT. Digital technologies for emotion-regulation assessment and intervention: a conceptual review. Clin Psychol Sci. (2022) 10(1):3–26. 10.1177/21677026211011982 35174006 PMC8846444

[95] Liddle J Burdon M Ireland D Carter A Knuepffer C Milevskiy N Balancing self-tracking and surveillance: legal, ethical and technological issues in using smartphones to monitor communication in people with health conditions. J Law Med. (2016) 24(2):387–97. 30137711

[96] Rashidisabet H Thomas PJ Ajilore O Zulueta J Moore RC Leow A. A systems biology approach to the digital behaviorome. Curr Opin Syst Biol. (2020) 20:8–16. 10.1016/j.coisb.2020.07.003

[97] Hilty DM Armstrong CM Luxton DD Gentry MT Krupinski EA. A scoping review of sensors, wearables, and remote monitoring for behavioral health: uses, outcomes, clinical competencies, and research directions. J Technol Behav Sci. (2021) 6:278–313. 10.1007/s41347-021-00199-2 PMC7819828 33501372

[98] Lee CC Chaspari T Provost EM Narayanan SS. An engineering view on emotions and speech: from analysis and predictive models to responsible human-centered applications | IEEE journals & magazine | IEEE Xplore. Proc IEEE. (2023) 111(10):1142–58.

[99] Teepe GW Lukic YX Kleim B Jacobson NC Schneider F Santhanam P Development of a digital biomarker and intervention for subclinical depression: study protocol for a longitudinal waitlist control study. BMC Psychol. (2023) 11(1):186. 10.1186/s40359-023-01215-1 37349832 PMC10288725

[100] Cychosz M Romeo R Soderstrom M Scaff C Ganek H Cristia A Longform recordings of everyday life: ethics for best practices. Behav Res Methods. (2020) 52:1951–69. 10.3758/s13428-020-01365-9 32103465 PMC7483614

[101] Dakanalis A Wiederhold BK Riva G. Artificial intelligence: a game-changer for mental health care. Cyberpsychology Behav Soc Netw. (2024) 27(2):100–4. 10.1089/cyber.2023.0723 38358832

[102] Diaz-Asper C Hauglid MK Chandler C Cohen AS Foltz PW Elvevåg B. A framework for language technologies in behavioral research and clinical applications: ethical challenges, implications, and solutions. Am Psychol. (2024) 79(1):79–91. 10.1037/amp0001195 38236217

[103] Smith E Storch EA Vahia I Wong STC Lavretsky H Cummings JL Affective computing for late-life mood and cognitive disorders. Front Psychiatry. (2021) 12:782183. 10.3389/fpsyt.2021.782183 ((Smith, Eyre) The PRODEO Institute, San Francisco, CA, United States(Smith, Eyre) Organisation for Economic Co-operation and Development (OECD), Paris, France(Smith) Department of Neurology Neurological Sciences, Stanford University, Stanford, CA, United). 35002802 PMC8732874

[104] Whelan R Barbey FM Cominetti MR Gillan CM Rosicka AM. Developments in scalable strategies for detecting early markers of cognitive decline. Transl Psychiatry. (2022) 12(1):473. 10.1038/s41398-022-02237-w 36351888 PMC9645320

[105] Milling M Pokorny FB Bartl-Pokorny KD Schuller BW. Is speech the new blood? Recent progress in AI-based disease detection from audio in a nutshell. Front Digit Health. (2022) 4(101771889):886615. 10.3389/fdgth.2022.886615 35651538 PMC9149088

[106] Nahar JK Lopez-Jimenez F. Utilizing conversational artificial intelligence, voice, and phonocardiography analytics in heart failure care. HEART Fail Clin. (2022) 18(2):311–23. 10.1016/j.hfc.2021.11.006 35341543

[107] Papakyriakopoulos O Choi ASG Andrews J Bourke R Thong W Zhao D Augmented datasheets for speech datasets and ethical decision-making. 2023 ACM Conference on Fairness, Accountability, and Transparency (2023). p. 881–904. Available online at: http://arxiv.org/abs/2305.04672 (Accessed May 16, 2024).

[108] Triantafyllopoulos A Kathan A Baird A Christ L Gebhard A Gerczuk M HEAR4Health: A blueprint for making computer audition a staple of modern healthcare. ArXiv Prepr ArXiv230110477. (2023).

[109] Frohlich H Bontridder N Petrovska-Delacreta D Glaab E Kluge F El Yacoubi M Leveraging the potential of digital technology for better individualized treatment of Parkinson’s disease. Front Neurol. (2022) 13:788427. 10.3389/fneur.2022.788427 35295840 PMC8918525

[110] Meltzer JA. Towards early prediction of Alzheimer’s disease through language samples. EclinicalMedicine. (2020) 29. 10.1016/j.eclinm.2020.100644 33294826 PMC7689272

[111] Adams WR. High-accuracy detection of early Parkinson’s disease using multiple characteristics of finger movement while typing. PLoS One. (2017) 12(11):e0188226. 10.1371/journal.pone.0188226 29190695 PMC5708704

[112] Nelson BW Allen NB. Extending the passive-sensing toolbox: using smart-home technology in psychological science. Perspect Psychol Sci. (2018) 13(6):718–33. 10.1177/1745691618776008 30217132

[113] Thomas J Arya L Hussain M Prasanna SRM. Speech act theory and ethics of speech processing as distinct stages: the ethics of collecting, contextualizing and the releasing of (speech) data. In: 2023 IEEE International Symposium on Ethics in Engineering, Science, and Technology (ETHICS). West Lafayette, IN, USA: IEEE (2023). p. 1–10. Available online at: https://ieeexplore.ieee.org/document/10154932/ (Accessed May 16, 2024).

[114] Corona Hernández H Corcoran C Achim AM de Boer JN Boerma T Brederoo SG Natural language processing markers for psychosis and other psychiatric disorders: emerging themes and research agenda from a cross-linguistic workshop. Schizophr Bull. (2023) 49(2):S86–92. 10.1093/schbul/sbac215 36946526 PMC10031727

[115] Isernia S Cabinio M Di Tella S Pazzi S Vannetti F Gerli F Diagnostic validity of the smart aging serious game: an innovative tool for digital phenotyping of mild neurocognitive disorder. J Alzheimers Dis. (2021) 83(4):1789–801. 10.3233/JAD-210347 34459394

[116] Batliner A Neumann M Burkhardt F Baird A Meyer S Vu NT Ethical awareness in paralinguistics: a taxonomy of applications. Int J Human Comput Interact. (2022):1–18.

[117] Sela Y Santamaria L Amichai-Hamburge Y Leong V. Towards a personalized multi-domain digital neurophenotyping model for the detection and treatment of mood trajectories. Sensors. (2020) 20(20):5781. 10.3390/s20205781 33053889 PMC7601670

[118] Anglade C Tousignant M Gaboury I. Rigorous qualitative research involving data collected remotely from people with communication disorders: experience from a telerehabilitation trial. Neurorehabil Neural Repair. (2022) 36(8):557–64. 10.1177/15459683221100489 35599591 PMC9373188

[119] Schuller DM Schuller BW. A review on five recent and near-future developments in computational processing of emotion in the human voice. Emot Rev. (2021) 13(1):44–50. 10.1177/1754073919898526

[120] Villongco C Khan F. Sorry I Didn’t hear you”. The ethics of voice computing and AI in high risk mental health populations. AJOB Neurosci. (2020) 11(2):105–12. 10.1080/21507740.2020.1740355 32228383

[121] Woodward K Kanjo E Brown DJ McGinnity TM Inkster B Macintyre DJ Beyond mobile apps: a survey of technologies for mental well-being. IEEE Trans Affect Comput. (2020) 13(3):1216–35. 10.1109/TAFFC.2020.3015018

[122] Nazer LH Zatarah R Waldrip S Ke JXC Moukheiber M Khanna AK Bias in artificial intelligence algorithms and recommendations for mitigation. PLoS Digit Health. (2023) 2(6):e0000278. 10.1371/journal.pdig.0000278 37347721 PMC10287014

[123] Jokinen K Declerck T. Researching Less-Resourced Languages—the DigiSami Corpus. (2018). p. 3382–6.

[124] Casteleyn L Dumez B Van Damme K Anwar WA. Ethics and data protection in human biomarker studies in environmental health. Int J Hyg Environ Health. (2013) 216(5):599–605. 10.1016/j.ijheh.2013.03.016 23660231

[125] Karabekmez ME. Data ethics in digital health and genomics. New Bioeth. (2021) 27(4):320–33. 10.1080/20502877.2021.1996965 34747348

[126] Lee CH Yoon HJ. Medical big data: promise and challenges. Kidney Res Clin Pract. (2017) 36(1):3–11. 10.23876/j.krcp.2017.36.1.3 28392994 PMC5331970

[127] Tiffin N George A LeFevre AE. How to use relevant data for maximal benefit with minimal risk: digital health data governance to protect vulnerable populations in low-income and middle-income countries. BMJ Glob Health. (2019) 4(2):e001395. 10.1136/bmjgh-2019-001395 31139457 PMC6509603

[128] Cummins N Scherer S Krajewski J Schnieder S Epps J Quatieri TF. A review of depression and suicide risk assessment using speech analysis. Speech Commun. (2015) 71:10–49. 10.1016/j.specom.2015.03.004

[129] Rubio DM Schoenbaum EE Lee LS Schteingart DE Marantz PR Anderson KE Defining translational research: implications for training. Acad Med. (2010) 85(3):470. 10.1097/ACM.0b013e3181ccd618 20182120 PMC2829707

[130] Bensoussan Y Sigaras A Rameau A Elemento O Powell M Dorr D Bridge2AI-Voice: An ethically-sourced, diverse voice dataset linked to health information. PhysioNet. (2025). Available online at: https://physionet.org/content/b2ai-voice/2.0.0/ (Accessed August 15, 2025).

[131] Awan SN Bahr R Watts S Boyer M Budinsky R Bensoussan Y. Evidence-Based recommendations for tablet recordings from the Bridge2AI-voice acoustic experiments. J Voice. (2024). Available online at: https://www.sciencedirect.com/science/article/pii/S0892199724002832 (Accessed March 14, 2025). 10.1016/j.jvoice.2024.08.029 PMC11922786 39306498

[132] Alberto IRI Alberto NRI Ghosh AK Jain B Jayakumar S Martinez-Martin N The impact of commercial health datasets on medical research and health-care algorithms. Lancet Digit Health. (2023) 5(5):e288–94. 10.1016/S2589-7500(23)00025-0 37100543 PMC10155113

[133] Daneshjou R Smith MP Sun MD Rotemberg V Zou J. Lack of transparency and potential bias in artificial intelligence data sets and algorithms. JAMA Dermatol. (2021) 157(11):1362–9. 10.1001/jamadermatol.2021.3129 34550305 PMC9379852

[134] Khan B Fatima H Qureshi A Kumar S Hanan A Hussain J Drawbacks of artificial intelligence and their potential solutions in the healthcare sector. Biomed Mater Devices. (2023) 1(2):731–8. 10.1007/s44174-023-00063-2 PMC9908503 36785697

[135] Aung YYM Wong DCS Ting DSW. The promise of artificial intelligence: a review of the opportunities and challenges of artificial intelligence in healthcare. Br Med Bull. (2021) 139(1):4–15. 10.1093/bmb/ldab016 34405854

[136] Ng MY Youssef A Miner AS Sarellano D Long J Larson DB Perceptions of data set experts on important characteristics of health data sets ready for machine learning: a qualitative study. JAMA Netw Open. (2023) 6(12):e2345892. 10.1001/jamanetworkopen.2023.45892 38039004 PMC10692863

[137] Evangelista EG Bélisle-Pipon JC Naunheim MR Powell M Gallois H, Bridge2AI-Voice Consortium, et al. Voice as a biomarker in health-tech: mapping the evolving landscape of voice biomarkers in the start-up world. Otolaryngol Head Neck Surg Off J Am Acad Otolaryngol Head Neck Surg. (2024) 171(2):340–52. 10.1002/ohn.830 38822764

[138] Lee H Friedman ME Cukor P Ahern D. Interactive voice response system (IVRS) in health care services. Nurs Outlook. (2003) 51(6):277–83. 10.1016/S0029-6554(03)00161-1 14688763

[139] Paik KE Hicklen R Kaggwa F Puyat CV Nakayama LF Ong BA Digital determinants of health: health data poverty amplifies existing health disparities—a scoping review. PLoS Digit Health. (2023) 2(10):e0000313. 10.1371/journal.pdig.0000313 37824445 PMC10569513

[140] Coppock H Jones L Kiskin I Schuller B. COVID-19 detection from audio: seven grains of salt. Lancet Digit Health. (2021) 3(9):e537–8. 10.1016/S2589-7500(21)00141-2 34303644 PMC8294804

[141] Health TLD. Do I sound sick? Lancet Digit Health. (2021) 3(9):e534. 34446262 10.1016/S2589-7500(21)00182-5

[142] Corple DJ Linabary JR. From data points to people: feminist situated ethics in online big data research. Int J Soc Res Methodol. (2020) 23(2):155–68. 10.1080/13645579.2019.1649832

[143] Montiel CJ Uyheng J. Foundations for a decolonial big data psychology. J Soc Issues. (2022) 78(2):278–97. 10.1111/josi.12439

[144] Cheung K Earp BD Patch K Yaden DB. Distinctive but not exceptional: the risks of psychedelic ethical exceptionalism. Am J Bioeth. (2025) 25(1):16–28. 10.1080/15265161.2024.2433421 39804307

[145] Garrison NA Brothers KB Goldenberg AJ Lynch JA. Genomic contextualism: shifting the rhetoric of genetic exceptionalism. Am J Bioeth AJOB. (2019) 19(1):51–63. 10.1080/15265161.2018.1544304 30676903 PMC6397766

[146] Murray TH. Is genetic exceptionalism past its sell-by date? On genomic diaries, context, and content. Am J Bioeth. (2019) 19(1):13–5. 10.1080/15265161.2018.1552038 30676900

[147] Shevchenko S Zhavoronkov A. The role of exceptionalism in the evolution of bioethical regulation. Camb Q Healthc Ethics. (2024) 33(2):185–97. 10.1017/S0963180123000336 37288492

[148] Alugubelli R. Exploratory study of artificial intelligence in healthcare. Int J Innov Eng Res Technol. (2016) 3(1):1–10.

[149] Mandl KD Manrai AK. Potential excessive testing at scale: biomarkers, genomics, and machine learning. JAMA. (2019) 321(8):739–40. 10.1001/jama.2019.0286 30735228 PMC7572222

[150] Panagoulias DP Virvou M Tsihrintzis GA. Regulation and validation challenges in artificial intelligence-empowered healthcare applications—the case of blood-retrieved biomarkers. In: Virvou M Saruwatari T Jain LC, editors. Knowledge-Based Software Engineering: 2022. Cham: Springer International Publishing (2023). p. 97–110. (Learning and Analytics in Intelligent Systems).

[151] Vazquez-Levin MH Reventos J Zaki G. Editorial: artificial intelligence: a step forward in biomarker discovery and integration towards improved cancer diagnosis and treatment. Front Oncol. (2023) 13:1161118. 10.3389/fonc.2023.116111 37064106 PMC10102612

[152] Gallois H Ivkovic L Evangelista E Bensoussan Y Dorr DA Elemento O “Low risk, high happiness”: a review of openly declared ethical and legal practices in voice biomarker health-tech start-ups. Health Care Anal. (2025). Available online at: 10.1007/s10728-025-00539-w (Accessed November 7, 2025). 41091343

[153] Blatter A Gallois H Evangelista E Bensoussan Y, Bridge2AI-Voice Consortium, Bélisle-Pipon JC. “Voice is the new blood”: a discourse analysis of voice AI health-tech start-up websites. Front Digit Health. (2025) 7:1568159. 10.3389/fdgth.2025.1568159 40510414 PMC12158947

[154] Wilson P. The voice and its metaphors. Aust Voice. (2004) 10:16–9.

[155] Cummins N Dineley J Conde P Matcham F Siddi S Lamers F Multilingual markers of depression in remotely collected speech samples: a preliminary analysis. J Affect Disord. (2023) 341:128–36. 10.1016/j.jad.2023.08.097 37598722

[156] Ritter E. Your voice gave you away: the privacy risks of voice-inferred information. Duke Law J. (2021) 71(3).

